# 25 Years of Cancer Immunoediting: Dendritic Cells and Macrophages Filled the Missing Gap

**DOI:** 10.3390/cancers18162672

**Published:** 2026-08-18

**Authors:** Vijay Kumar, John H. Stewart IV

**Affiliations:** Department of Surgery, Laboratory of Tumor Immunology and Immunotherapy, Medical Education Building-C, Morehouse School of Medicine, 720 Westview Drive, Atlanta, GA 30310, USA; jstewart@msm.edu

**Keywords:** cancer, cancer immunosurveillance, cancer immunoediting, cancer immunotherapy, TAM, DCs

## Abstract

In 1909, Paul Ehrlich first suggested that the immune system is critical for suppressing carcinogenesis and cancer growth, which led to the introduction of cancer immunosurveillance by Sir MacFarlane Burnet in 1960. Burnet’s cancer immunosurveillance was further supported and elaborated by Thomas Lewis in 1982, who noted, for example, the limitations of investigating cancer immunosurveillance in available experimental models, except for virus-induced cancers. The concept of cancer immunosurveillance in 2001 was revised to cancer immunoediting (the immunoediting term was introduced by Dr. Rober Schreiber), based on findings that the immune system not only controls tumorigenesis and tumor growth but also tumor quality/grade or immunogenicity. Advances in immunology, including the identification of target organs and the local immune system, and the identification of novel innate immune cells regulating the adaptive immune response, have further advanced the understanding of cancer immunosurveillance and immunoediting. The current article discusses the evolution of the cancer immunosurveillance hypothesis into cancer immunoediting. At the time of the introduction of cancer immunoediting, the direct role of dendritic cells (DCs) in tumor immunity and immunoediting was not much explored. Similarly, the concept of tumor-associated macrophages (TAMs) and their role in cancer immunosurveillance and immunoediting was not much studied. The current article discusses the evolution of DCs and TAMs in the context of tumor immunity, cancer immunoediting, and immunotherapies specifically targeting these innate immune cells.

## Simple Summary

The immune system plays a critical role in carcinogenesis and metastasis. The importance of the immune system in fighting cancer was first suggested by Nobel laureate Paul Ehrlich in 1909. Moreover, in 1960, another Nobel Prize winner, Sir MacFarlane Burnet, proposed the concept of cancer immunosurveillance, suggesting that the immune system continuously patrols the cancer environment. Thomas Lewis, in 1982 further supported cancer immunosurveillance. Introduction of cancer immunoediting in 2001 by Dr. Schreiber revolutionized the cancer immunology research. Cancer immunoediting suggested that the immune system not only controls carcinogenesis but also the tumor quality and antigenicity. However, at the introduction of cancer immunoediting, the role of macrophages, including resident tissue macrophages and dendritic cells (DCs), which are critical innate immune cells with a regulatory impact on adaptive immunity, was unclear. Therefore, the current article discusses macrophages and DCs in the context of cancer immunoediting, including filling immunological gaps that were understudied and the development of macrophage- and DC-based cancer immunotherapies.

## 1. Introduction

**Figure 1:**
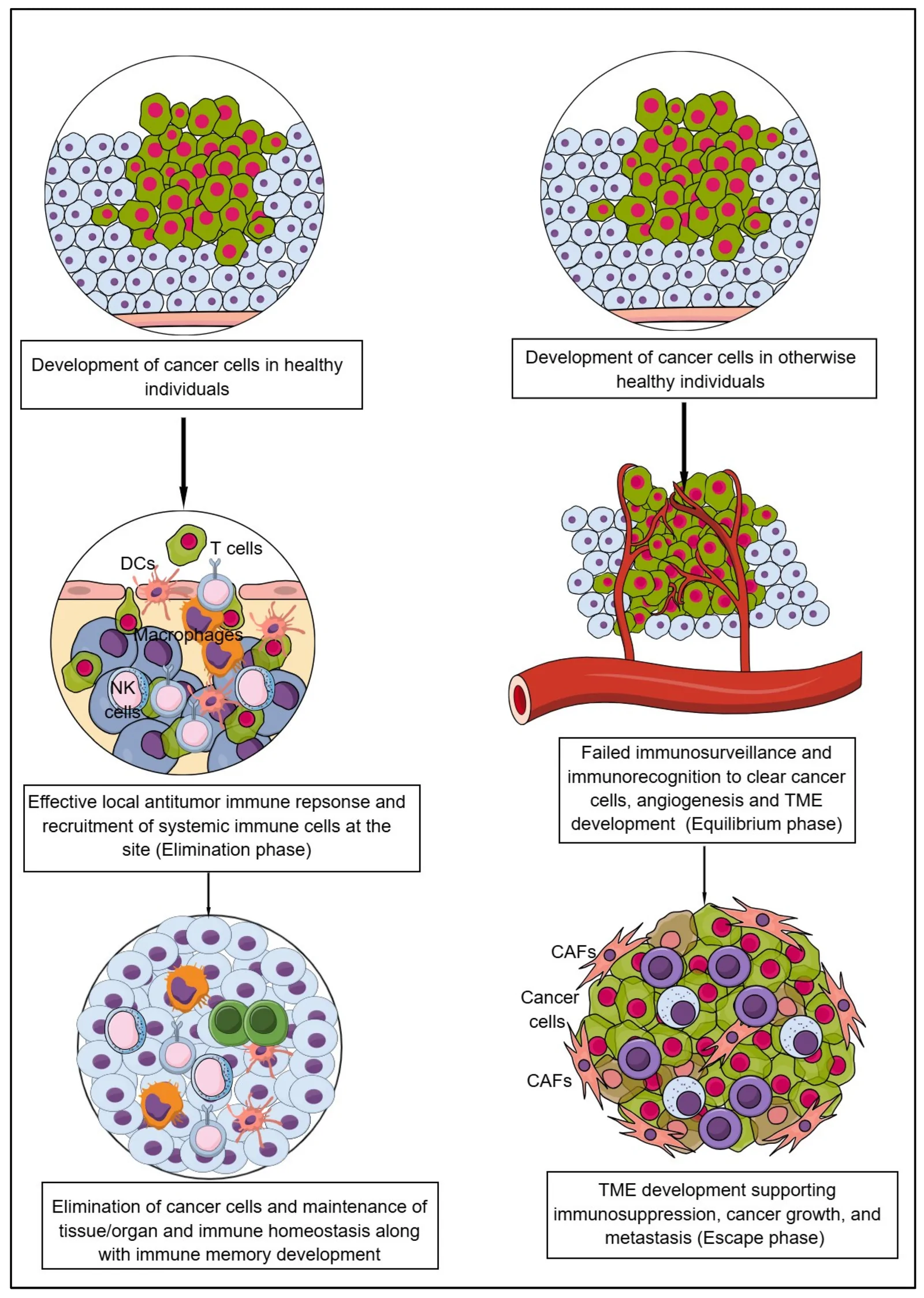
Basis of cancer immunosurveillance and immunoediting. The transformed cancer cells are cleared by local and migrating immune cells in healthy individuals (who never develop clinically diagnosed cancer) due to a strong elimination phase to maintain tissue/organ homeostasis along with immune homeostasis. Moreover, during this process, immune cells develop immune memory, like trained immunity (in case of innate immune cells) and classical immune memory (comprising memory T and B cells). On the other hand, failure of elimination phase in patients with clinically diagnosed cancer leads to equilibrium and the escape phase, which are responsible for cancer growth and proliferation, leading to TME formation. For example, CAFs support pro-tumorigenic signaling mechanisms, tumor progression, and repression of antitumor immune response. TME development backs the *escape phase* of the cancer immunoediting. Kindly see the text for details.

Almost every organ and tissue is continuously monitored by local residential and mobile/circulating immune components, such as innate and adaptive immune cells and the factors (cytokines, chemokines, interferons (IFNs), and complement proteins) they secrete. These secreted factors further direct resident and migrating immune cells to maintain immune homeostasis and metabolism, along with local tissue or organ homeostasis (Figure 1) [1,2,3,4]. Moreover, these tissue-resident immune cells (TRICs), including innate [macrophages, dendritic cells (DCs), and natural killer (NK) cells] and adaptive immune cells (T and B cells), also develop immune memory. For example, innate immune memory (trained immunity) and conventional adaptive immune memory are also critical for maintaining tissue and organ homeostasis in addition to immune homeostasis (Figure 1) [5,6,7,8,9]. Thus, naïve and memory-immune cells are both critical for tissue and organ homeostasis (Figure 1) [5].

**Figure 2:**
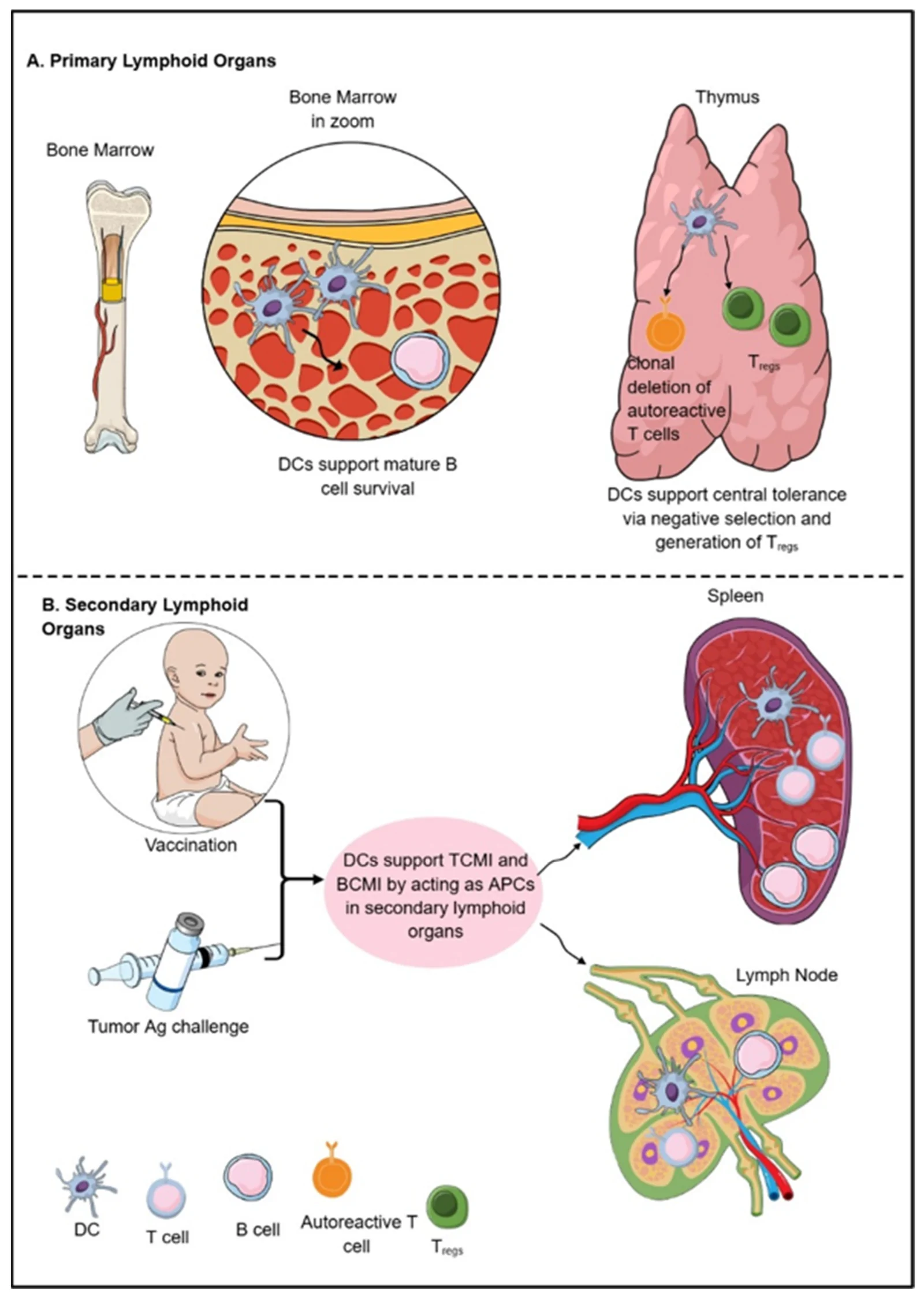
DCs are present in primary and secondary lymphoid organs (PLOs and SLOs) and serve as critical APCs. (**A**). DCs are present in PLOs, like BM and the thymus. In the BM, DCs support survival of mature B cells, and in the thymus they are critical to maintaining central tolerance by supporting clonal deletion of autoreactive T cells and generation of T_regs_, which is a critical process to avoid self-attacking autoimmune T cells, responsible for several autoimmune diseases. (**B**). DCs are also present in SLOs, like the spleen and LNs. Their presence in SLOs is critically needed for generating a potent immune response against infections, vaccines, and tumor Ags. In SLOs, DCs serve as potent APCs to generate TCMI and BCMI against invading infections and vaccines, including tumor Ags.

DCs are localized to central/primary lymphoid organs (CLOs/PLOs), such as bone marrow (BM) and the thymus, and secondary lymphoid organs (SLOs), such as lymph nodes (LNs) and the spleen (Figure 2) [10]. In the BM, DCs critically support mature B cell survival, and in the thymus they mediate central tolerance via negative selection or clonal deletion of autoreactive T cells, and induction of regulatory T cells (T_regs_), which suppress self-reactive T cells (Figure 2). In SLOs, by serving as potent antigen (Ag)-presenting cells (APCs), DCs help to initiate potent T cell-mediated immunity (TCMI) that also regulates T cell-dependent B cell-mediated immunity (BCMI) and antibody (Ab) generation during infection or upon Ag challenge, such as vaccination or tumor Ag encounter (Figure 2) [10,11,12]. In addition to lymphoid organs, DCs are present in almost all organs, such as epithelial surfaces, including stratified squamous cell epithelia (vagina, cervix, anus, pharynx, and esophagus) in direct contact with the external environment and all other organs, such as lungs, liver, heart, kidneys, brain, and gastrointestinal tract (GIT) [10]. Similarly, macrophages have been recognized in almost every organ and are named depending on their target organ location and regulate local tissue/organ homeostasis and disease pathogenesis [13,14,15,16]. These different target organ macrophages exhibit different gene expression and transcriptional regulatory pathways (chemokine receptor expression, toll-like receptor (TLR), C-type lectin receptor (CLR) domain members, and efferocytic receptors), governing their functions [17]. Therefore, DC and macrophage dysregulation are critical for cancer pathogenesis. To survive, grow, and metastasize, cancer cells must evade local and systemic immunorecognition, a process hypothesized in 2001 as cancer immunoediting by Dr. Robert Schreiber, following a major revision of the cancer immunosurveillance hypothesis (Figure 1) [18,19]. Therefore, the current article discusses the evolution of the concept of cancer immunosurveillance, leading to the development of the cancer immunoediting concept in 2001, and how DCs and monocytes/macrophages, including tumor-associated macrophages (TAMs), have marked the unknown issues of cancer immunoediting and led to the development of cancer immunotherapies targeting DCs and TAMs.

## 2. Evolution of Cancer Immunosurveillance Hypothesis to Cancer Immunoediting

Nobel Laureate Paul Ehrlich, over a century ago in 1909, first predicted that the immune system is critical for suppressing carcinoma growth [20]. According to him, in the absence of an intact immune system, long-living organisms, including humans, would more frequently develop tumors and succumb to death (Figure 1) [19,20,21]. The cancer immunosurveillance concept/hypothesis was proposed by the Nobel Prize winner (1960, for predicting acquired immune tolerance) Sir Frank Macfarlane Burnet. Sir Macfarlane Burnet had two sides to his opinion. On one hand, he stated that “there is little ground for optimism about cancer.” However, on the other hand, he was optimistic about preventing cancer through immunological methods (cellular immune system). His optimism about immune-mediated cancer intervention was based on findings indicating a strong association between viral infections and neoplasia, and on more effective chemotherapeutics exhibiting tissue interactions at immunological levels [22,23]. Thus, immunological aspects of cancer biology, or neoplasia, were elaborated to “cancer immunosurveillance” by Sir Macfarlane Burnet and other researchers’ observations [24,25].

Later, in 1982, Dr. Thomas Lewis further highlighted the limitations of studying cancer immune surveillance (immunosurveillance) in available experimental animals, except for virus-induced cancers [26]. However, the presence of a cluster of neoplastic cells in the biopsies of older, healthy individuals who never develop cancer indicates that neoplastic cells are regularly produced in the body, but are continuously recognized and cleared by the vigilant immune system (Figure 1) [26]. People who fail to eliminate developing neoplastic/cancer cells through mechanisms such as immunosurveillance and immunorecognition develop cancers more easily than otherwise healthy individuals (Figure 1) [26]. This initial cancer immunosurveillance theory of Burnet and Thomas, which suggested the critical role of thymus-derived T cells in the process, was challenged by the emergence of contradictory findings from athymic nude mice. The data from athymic nude mice indicated that thymus-dependent immunity plays no significant role in the cancer immunosurveillance mechanism that protects against tumor development [27,28,29].

However, during the same period, researchers showed that a cooperative innate and adaptive immune response prevents the growth of injected lymphoma cells in mice [30]. Even early researchers correlating spleen immune cells, inflammation, and cancer pathogenesis suggested pro-tumoral effects of the immune system per their observations, indicating overaccumulation of lymphocytes and cells of the reticular endothelial system (RES, currently known as the mononuclear phagocytic system or MPS), which was against Burnet’s cancer immunosurveillance hypothesis [31,32,33,34]. Moreover, subsequent studies indicated that nude mice do not completely lack functional T cells, and that these T cells have a limited T cell receptor (TCR) repertoire, reflecting the in vivo expansion of a relatively small number of functional precursors [35,36,37]. Later studies have suggested the protective antitumor functions of IFN-γ and perforin, which are mainly secreted by cytotoxic T and NK cells [18,38,39]. Now we know that athymic/nude mice retain functional B cells and an intact innate immune system, including macrophages and NK cells, which protect against developing tumors [40,41]. Moreover, use of recombinase activating gene-2 (RAG-2) knockout mice, lacking peripheral mature αβ T and B cells, further strengthened the protective role of adaptive immunity against chemical (methylcholanthrene or MCA) carcinogenesis [18,42]. Therefore, initially cancer immunosurveillance did not recognize the importance of the innate immune system in the process, as it was in its infancy.

Previous articles on cancer immunosurveillance and immunoediting by pioneers in the field, including Dr. Robert Schreiber, have discussed in detail the roles of NK cells, IFN-γ, perforin, Fas/Fas ligand (FasL), IFNs, and T cells in these processes [20,21,43,44,45,46,47,48,49]. The current article will not discuss T cells and NK cells, along with immunotherapies targeting them, as it focuses on DCs and macrophages in cancer immunoediting and their targeting to develop cancer immunotherapies. Moreover, antitumor and pro-tumorigenic effects of IFNs depend on their types [type 1 (IFN-α, IFN-β, IFN-ε, IFN-κ, and IFN-ω), type 2 (IFN-γ), and type 3 (IFN-λ)], cellular targets, and duration of activity, as discussed elsewhere [49,50,51].

It is interesting to note that during this era, the field of innate immunity was in its infancy, despite the discovery and description of phagocytosis and phagocytes by Elia Metchnikoff in 1882, who received the Nobel Prize in 1908 for this work [52]. Moreover, it took almost another century for the discovery of toll-like receptor 4 (TLR4) as a pattern-recognition receptor (PRR) for the Gram-negative bacterial lipopolysaccharide (LPS) in human immune cells, such as macrophages, by the research group led by Charles A. Janeway Junior in 1997 [53]. Before the discovery of TLR4 in his laboratory, Charles A. Janeway Jr. proposed the pattern recognition theory at the Cold Spring Harbor Symposium on Immune Recognition in 1989. This hypothesis lecture provided a conceptual framework for our current understanding of innate immune recognition and the regulation of the adaptive immune response via innate immune responses [54,55].

The discovery of TLR4 first filled the long-standing gap in the immunologic recognition of pathogens, their clearance, and innate immune system-mediated regulation of adaptive immunity [53,56]. Now, we know that TLRs not only recognize microbial or pathogen-associated molecular patterns (MAMPs or PAMPs), but also death/damage-associated molecular patterns (DAMPs), thereby generating an inflammatory innate immune response [56,57,58,59,60]. These TLRs are also expressed by adaptive immune cells (T and B cells) and regulate their immunological functions, which are critical for generating antitumor immunity, as discussed elsewhere [61]. Thus, before the discovery of TLRs and their immunological contributions, the immunological control or elimination of tumors was considered only thymus- or T cell-dependent [24,62,63]. Hence, the discovery of phagocytes (monocytes and macrophages), phagocytosis, and TLRs has changed the over half-century-old cancer immune surveillance/immunosurveillance hypothesis proposed by MacFarlane Burnet and later supported by Thomas Lewis in 1982 [26].

For example, the current/revised cancer immunosurveillance hypothesis, which has now been updated to *cancer immunoediting* in 2001, is based on the findings that the immune system not only controls the tumor size/quantity but also the tumor quality/grade or immunogenicity due to the involvement of both (innate and adaptive immune) components [18,19]. This is because cancer immunosurveillance describes only one side of the cancer–immune system relationship. For example, the 2001 cancer immunosurveillance hypothesis evolved in response to the findings that innate immune cells, such as NK and NKT cells, and their secretory products (IFN-γ and IL-12) provide differential tumor surveillance and also exert antimetastatic effects in gene-targeted and specific lymphocyte-deficient mice [64,65,66].

A study from the laboratory of Dr. Schreiber in 1994, utilizing a neutralizing monoclonal Ab against murine IFN-γ, further suggested that IFN-γ exerts a direct effect on tumor cell immunogenicity, which is critical for their recognition and elimination [38]. For example, according to Dr. Shcreiber’s research findings, the transplantation of IFN-γ-insensitive Meth A cells (a BALB/c mouse-derived fibrosarcoma cell line) to BALB/c mice exhibits higher tumorigenicity than control Meth A cells. LPS treatment of tumor-bearing mice did not induce rejection of IFN-γ-insensitive Meth A cells but eliminated control tumors (regular IFN-γ-sensitive Meth A cells) [38]. Interestingly, LPS induces IFN-γ production from both innate and adaptive immune cells, such as macrophages, NK cells, and T cells, which can be further enhanced by IL-2 and IL-12 [67,68]. LPS is a well-known TLR4 ligand; thus, the discovery of TLR4 has solved the mystery of LPS-mediated IFN-γ production by utilizing the downstream IL-1 receptor-associated kinase 1 (IRAK) and NF-κB signaling pathway (TLR4/IRAK/NF-κB axis), along with producing cytokines/molecules, which stimulate/enhance IFN-γ production [69]. IFN regulatory factor 5 (IRF5) expression, which is upregulated during TLR4 signaling, has shown a direct association with IFN-γ levels [70]. IRF5 signaling in CD4^+^T cells also induces IFN-γ production, and human peripheral and splenic CD4^+^T cells express TLR4, which is further influenced by macrophage migration factor (MIF) [71,72]. BALB/c mice CD4^+^CD45b^low^CD4^+^T cells express TLR4 and can respond to LPS-mediated TLR4 stimulation to produce IFN-γ [61].

Moreover, utilization of perforin-deficient mice further suggested that cytotoxicity mediated by perforin (mainly secreted by cytotoxic T and NK cells) is critical for surveillance of spontaneous lymphoma, suggesting the critical role of NK and cytotoxic T cells during viral and chemical carcinogenesis, including lymphomagenesis [39,73,74]. The local gamma–delta T cells (γδT cells) have also been shown to play a critical role in combating cutaneous carcinogenesis [75]. Furthermore, cancer cells, such as human breast cancer cells, overexpress IRF2, which is antagonistic to IRF1. IRF2 expression in cancer cells promotes their growth and inhibits type 1 IFN (IFN-β) production and thus the pro-inflammatory antitumor immune response [76,77,78]. The IRF2-expressing tumor CD8^+^T cells exhibit exhaustion in response to type 1 and 2 IFNs and promote tumor growth and immune evasion [79]. However, there is another side: tumors formed in mice without an intact immune system, such as athymic/nude mice, possess more immunogenic properties and are therefore called “unedited,” whereas tumors derived from mice with an intact immune system are called “edited” as a result of potential immune selection [18,19]. In humans (with an intact immune system) with primary and treatment-naïve tumors, the immune system restricts cancer cells expressing immunogenic Ags from the clonal population [80]. For example, immunocompetent cancer patients (treatment-naïve and with an intact immune system) have lower overall mutational burdens and lower clonal mutational burdens than patients with cancer having low-immune-infiltrate tumors/TME from immunosuppressed patients. Thus, immunoediting restricts clonal neoAgs in primary treatment-naïve human tumors.

Thus, these immunological findings, in the context of cancer pathogenesis, led to the evolution of the cancer immunosurveillance hypothesis. This evolved cancer immune surveillance hypothesis comprises the involvement of innate and adaptive immune systems in the process of cancer–immune system face-off through a process called cancer immunoediting. Cancer immunoediting comprises three different stages or 3Es: (1) *Elimination*, (2) *Equilibrium*, and (3) *Escape*, as discussed in detail elsewhere by Dr. Robert Schreiber and colleagues [20,81]. In brief, *the elimination* phase represents the original concept of cancer immunosurveillance, in which successful clearance of cancer cells occurs without involving subsequent phases (Figure 1). The *equilibrium* phase is the longest of the three phases and may last for years due to a failed elimination phase. During *the equilibrium* phase, a continuous low-grade antitumor immune response leads to the emergence of cancer cells with unstable genomes and high mutation potential, forming a cancer bed or TME, with an increasing population of cancer cells exhibiting low antigenicity. The third *escape* phase involves the growth of cancer cells with low antigenicity and their spread to distant organs via metastasis (Figure 1).

It is worth noting that immunosuppressive pre-metastatic niches (PMNs) in distant organs develop via systemic and local PMN immunosuppression to support metastasis, as discussed in detail elsewhere [82,83,84]. Hence, the foundation of the cancer immunoediting concept is that the immune system not only fights cancer cells to prevent tumor development but also shapes tumor immunogenicity, indicating its dual role (anti- and pro-) in carcinogenesis and metastasis. Since 2001, great advances have occurred in the field of innate immunity and its regulatory mechanisms, with the potential to refine and explore unsolved immunological mysteries that could not be addressed earlier in the processes of cancer immunosurveillance and cancer immunoediting. Therefore, the current article will discuss the impact of macrophages and DCs in cancer immunosurveillance, immunoediting, and the development of DC- and macrophage-based immunotherapies.

## 3. Evolution of Concept of TME and Tumor-Associated Macrophages (TAM), and Their Targeting

The foundation of the TME was laid in 1889, when Stephen Paget published the seed–soil hypothesis to understand cancer initiation and progression [85,86,87,88]. However, it was revisited by several researchers from the mid-seventies of the 20th century and onwards to utilize TME (a highly structured tumor ecosystem) for exploring and understanding the cellular environment of the cancerous tissue, which not only comprises cancer cells but also local cancer-associated fibroblasts (CAFs), epithelial cells, and endothelial cells, along with local and infiltrated immune cells and their extracellular and secretory products, such as extracellular matrix, cytokines, chemokines, growth factors, enzymes, and metabolites [86,89,90,91]. The initiation and formation of the TME create a tumor-supportive environment; for example, the transformation of local quiescent fibroblasts to CAFs triggers a wide variety of pro-tumorigenic signals, supporting tumor progression and shaping the surrounding pathological stroma by remodeling tissue architecture and repressing the local immune response [92,93]. Thus, to survive, grow, proliferate, and metastasize, cancer cells, along with transforming local fibroblasts into CAFs, also transform other local precursors, such as mesenchymal stem cells (MSCs), endothelial cells, and myoepithelial cells, into CAFs. CAFs secrete several immune mediators (cytokines and chemokines; IL-7, IL-8, TGF-β, TNF-α, CCL5, CXCL9, CXCL10, and CXCL12), angiogenesis factors (vascular endothelial growth factor or VEGF), and matrix metalloproteases (MMPs) to support a chronic pro-inflammatory tumor-supportive environment along with supporting tumor angiogenesis and epithelial cell–mesenchymal cell transition (EMT) [94,95,96,97]. Hence, initiation and establishment of the TME represent the initiation and establishment of the equilibrium phase of cancer immunoediting, in which mutant and genetically unstable cancer cells continue to develop with a greater tendency to escape or evade local and systemic immunity.

In the TME, mitochondrial transfer, such as the transfer of mutated mitochondrial DNA (mtDNA) from cancer cells to tumor-infiltrating leukocytes (TILs), also induces immune evasion by blocking mitophagy through the transfer of mitophagy-inhibitory molecules [98]. For example, T cells carrying cancer mitochondria exhibit altered metabolism or immunometabolism leading to their exhaustion or senescence. This reduces their antitumor activity, and the presence of mtDNA mutations in tumor tissue is a poor prognostic factor for ICIs in patients with melanoma and non-small cell lung cancer (NSCLC) [98]. Moreover, in the TME, not only cancer cells but also local immune cells undergo epigenetic and metabolic reprogramming (immunometabolic reprogramming) to support cancer cell growth, division, proliferation, and metastasis, leading to the induction of the immune escape phase (third E) of the cancer immunoediting process [99,100,101,102,103,104,105,106]. It is interesting to note that the TME varies with the target organ cancer, determining whether immune cell activation and infiltration are tumor-suppressive or tumor-supportive [107].

**Figure 3:**
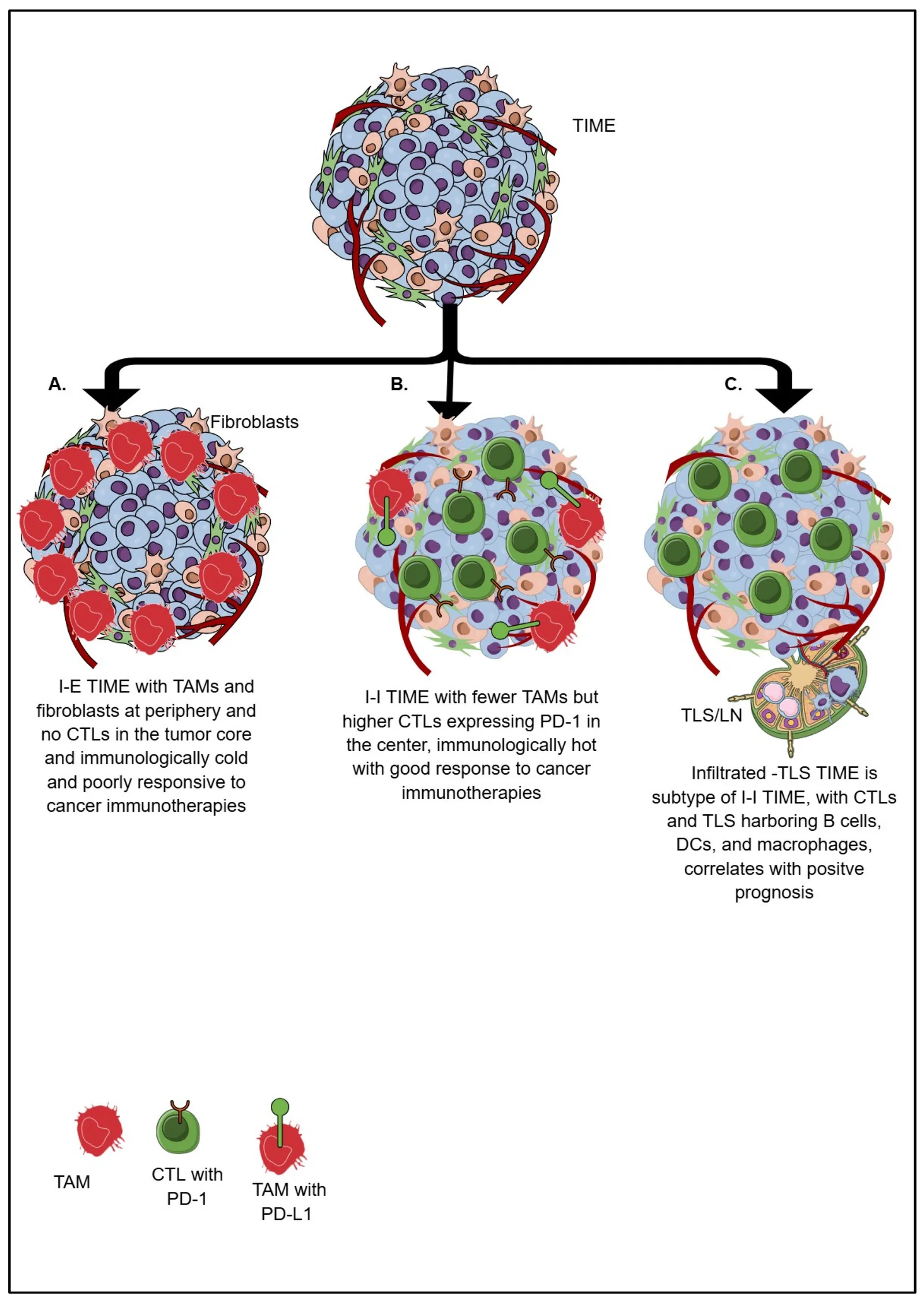
TIME classification depending on CTL infiltration. (**A**). I-E TIME lacks CTLs in the tumor core. These are poorly responsive to cancer immunotherapies and are called “Immunologically cold” tumors. (**B**). I-I TIME has higher CTLs in the core as compared to I-E TIME. Their CTLs express PD-1 and are highly responsive to cancer immunotherapies. They are “immunologically hot”. (**C**). Infiltrated-TLS TIME is a subtype of I-I TIME and has a higher number of CTLs with TLS having B cells, DC, and macrophages. These tumor types exhibit positive prognosis.

Local and infiltrated immune cells in the TME comprise its immune environment, which is called the tumor immune microenvironment (TIME), and determines the efficacy of immunotherapies (Figure 3). Techniques such as high-resolution single-cell RNA sequencing, flow cytometry, and intra-vital microscopy have been developed to identify the composition, function, and location of immune cells within the TME/TIME. Generally, the TIME is classified as a pro-tumor or immunosuppressive TIME and an antitumor/pro-inflammatory/immunostimulatory TIME. However, it has recently been classified on the basis of the presence/infiltration of cytotoxic T lymphocytes (CTLs) into the core of the TIME (Figure 3) [108]. For example, TIMEs lacking CTLs in their core are called infiltrated-excluded (I-E) TIMEs (Figure 3). I-E TIMEs, which are common in various epithelial cancers, such as colorectal carcinoma, melanoma, and pancreatic ductal carcinoma (PDAC), have CTLs localized to the border of the tumor mass in the invasive margin or caught in fibrotic nests [108]. Moreover, tumor-associated macrophages (TAMs) along the tumor margins also play a crucial role in preventing CTL infiltration into the TIME (Figure 3) core and their (TAMs and other myeloid cells) deletion enhances anticancer immunity associated with gemcitabine treatment in mice with PDAC [109,110]. On the other hand, infiltrated-inflamed (I-I) TIMEs are “immunologically hot” and have higher numbers of CTLs in their core, which express PD-1 (Figure 3). In addition, I-I TIMEs also have PD-L1-expressing leukocytes in the core and cancer cells also express PD-L1 [108]. A subclass of the I-I TIME is called the TLS-TIME, which develops tertiary lymphoid structures (TLSs), having a cellular composition similar to that of LNs (Figure 3). The details of the TIME in establishing tumors, its metastasis, and its genotype and phenotypic regulations have been discussed in detail elsewhere [108]. Therefore, the evolution of the TME/TIME and the recognition of macrophages within it led to the introduction of TAMs, which are critical for tumor angiogenesis/neoangiogenesis and associated immunosuppression, and targeting them can block these processes to target cancer (Figure 2) [111,112,113].

### 3.1. Macrophages and TAMs in TME and Cancer Immunoediting

Macrophages are myeloid innate immune cells with phagocytic and APC capacity, which are ubiquitous in all tissues and organs and have been observed in all solid tumors [52,114,115]. It is interesting to mention that in their basal/homeostatic state, resident tissue macrophages (RTMs) exhibit a high degree of diversity in their morphologies, transcriptional profiles, and anatomical locations and functional capabilities due to their interaction with cell types comprising that tissue structure [116]. However, details about their origin and function across different tumor types were unclear at the time of the first proposal of cancer immunoediting and remain under investigation [117]. Interestingly, earlier studies have reported the anti- and pro-tumor roles of macrophages *in vitro* (in which isolated macrophages from immunized mice exhibit antitumor activity by killing cancer cells) and their high infiltration into the TME, which develops as a result of chronic inflammation [31,118,119,120]. For example, macrophages isolated from patients with malignant tumors exhibit higher phagocytic activity [121]. However, another study showed no effect of bronchogenic carcinoma on the phagocytic activity of circulating monocytes despite their increased circulation [122]. Thus, the role of macrophage-mediated phagocytosis during cancer pathogenesis and metastasis was initially unclear due to limited studies. Future studies were needed to understand the direct association of phagocytic functions of TME macrophages, which phagocytose cancer cell debris and/or apoptotic cancer cells.

Interestingly, circulating monocytes derived from patients with bronchogenic carcinoma have decreased chemotaxis compared to the control group. Thus, the pro- and antitumor actions of macrophages were known at the origin of the cancer immunosurveillance hypothesis and during its evolution into the cancer immunoediting hypothesis in 2001. However, the immunological aspects of local macrophages in the TME and the emergence of TAMs were yet to come [123,124,125].

**Figure 4:**
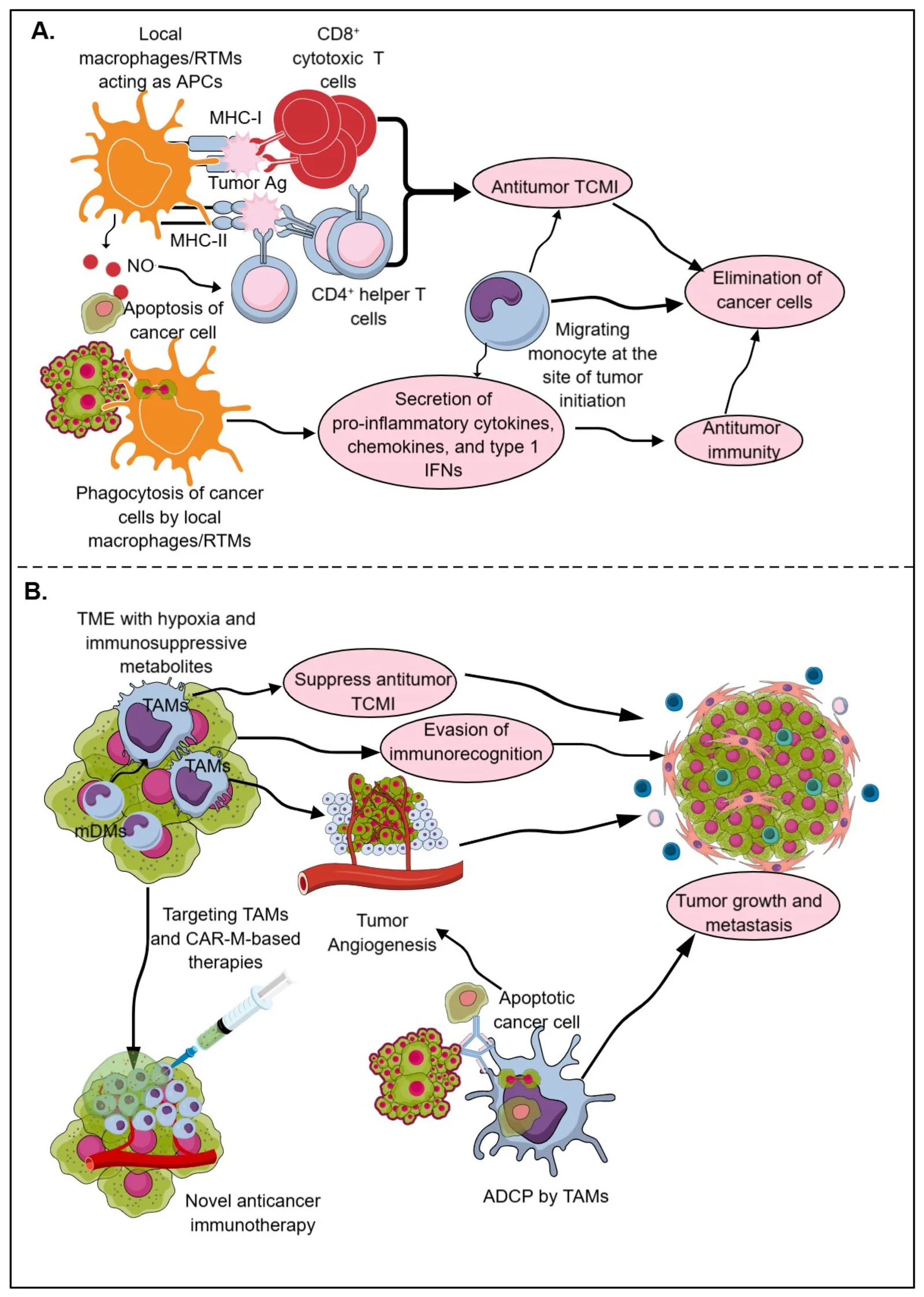
Macrophages exert anti- and pro-tumorigenic functions. (**A**). Local macrophages exert antitumor activity through their innate immune functions, such as phagocytosis, Ag presentation to helper and cytotoxic T cells via MHC-II and MHC-I molecules, and secreting several pro-inflammatory cytokines, chemokines, and IFNs, which further support antitumor immunity to support the elimination phase of the cancer immunoediting. Moreover, NO^.^ released from macrophages serving as APCs diffuses into nearby cancer cell and induces its apoptosis, which are cleared by efferocytosis during early carcinogenesis to support elimination phase. Moreover, migrating mDMs also support antitumor TCMI and secrete antitumor pro-inflammatory cytokines, chemokines, and IFNs to support antitumor immunity during the elimination phase. (**B**). However, in people who fail to eliminate cancer cells during early carcinogenesis, rapidly dividing cancer cells create a hypoxic environment and generate immunosuppressive metabolites, and factors, which suppress the antitumor activity of local and migrating macrophages. These macrophages are called TAMs. TAMs help in creating an immunosuppressive TME, support tumor angiogenesis, help in immunoevasion by cancer cells, and ADCP of apoptotic cancer cells by TAMs, which is immunosuppressive and further supports tumor angiogenesis. Thus, targeting TAMs have led to the development of macrophage-based immunotherapies, including CAR-Ms. Details are mentioned in the text.

Local macrophages/RTMs, depending on their phenotype and function, like other immune cells, exert antitumor activity through phagocytosis, Ag presentation, and secreting pro-inflammatory cytokines (TNF-α, IL-1β, IL-6, and IL-8), chemokines (CXCL-1, 2, 3, 5, 8, 9, and 10) and type 1 IFNs and IFN-γ in people with an intact immune system capable of exhibiting the elimination phase of cancer immunoediting (Figure 4A) [126]. Moreover, tumor-infiltrating monocytes also exert antitumor actions by secreting reactive oxygen species (ROS), including nitric oxide (NO), by undergoing metabolic reprogramming utilizing the L-arginine signaling pathway and differentiating into pro-inflammatory antitumor M1 macrophages, which, along with secreting pro-inflammatory cytokines, also activate antitumor CD4^+^ and CD8^+^T cells (Figure 4A) [127,128]. However, this antitumor activity of RTMs might be altered by the metabolites (lactate) and factors (TGF-β, IL-10) released from rapidly growing and proliferating cancer cells [129]. These cancer cells also create a hypoxic environment, leading to the equilibrium phase of cancer immunoediting, with the development or migration of tumor-promoting monocyte-derived macrophages (mDMs) in the TME (Figure 4A,B) [130,131,132].

In 1987, Hibbs et al. showed that macrophages utilize L-arginine to produce NO^.^, which exerts direct cytotoxic action on phagocytosed cancer cells [133,134]. Later, other researchers also showed the involvement of macrophage NO^.^ in antitumor activity and in the regulation of immune functions of other immune cells, such as lymphocyte proliferation (Figure 4A) [135,136]. Moreover, the pro-inflammatory M1 and anti-inflammatory M2 macrophage concepts, which depend on L-arginine metabolism and NO^.^ production, nitric oxide synthase (NOS), and arginase activity, also emerged in 2000, before the cancer immunoediting hypothesis, and can influence the Th1/Th2 immune response [41].

The NO^.^ produced by macrophages following Ag presentation to Th1 cells suppresses Th1 and Th2 proliferation [137]. The IFN-γ secretion from Th1 cells induces the production of NO^.^ by macrophages during Ag presentation to Th1 cells. Thus, NO^.^ produced from macrophages might exert direct cytotoxic action on cancer cells but suppress Th1 and Th2 cell proliferation in the TME. The macrophage-derived NO^.^ suppresses Th1 cell proliferation in the TME at very high concentrations. However, at low or physiological concentrations, it induces adhesion molecule (vascular cell adhesion molecule-1 or VCAM-1) expression on tumor vessels that supports T cell extravasation in the TME and tumor rejection, supporting the elimination phase of cancer immunoediting [138]. For example, iNOS-mediated NO^.^ production in macrophages in response to recognition of Ag during Ag presentation exerts anticancer activity. During Ag presentation, NO^.^ from the macrophage diffuses into neighboring cancer cells and induces their apoptosis (Figure 4A) in response to the activation of the mitochondrial pathway due to accumulation of peroxynitrite [139]. The details of NO^.^ in cancer pathogenesis and lymphocyte function regulation have been discussed elsewhere [140,141].

It is interesting to mention that depending on their location, RTMs might promote initial carcinogenesis. For example, a murine study using a transplantable p53-null model of early progression of breast cancer has indicated that early recruitment and activation of macrophages to ductal hyperplasia with a high tumor-forming potential supports tumor progression (premalignant lesions to invasive cancer) [142]. Similarly, RTMs aid in pro-tumorigenesis during the initiation phase of NSCLC as they accumulate close to tumor cells and promote the EMT process and cancer invasiveness. Furthermore, RTMs induce potent T_reg_-mediated immunosuppression during the initiation phase of NSCLC, which suppresses NK- and cytotoxic T cell-mediated killing and clearance of cancer cells [143].

The accumulation of RTMs close to cancer cells occurs up to day 15 after tumor seeding, before redistributing to the periphery of the tumor lesions, resembling human granuloma lesions; similar findings were reported in a genetically engineered mouse model of NSCLC [143,144,145,146]. This study has identified the existence of homologous lung macrophages between mice and humans, providing an opportunity to identify the lineage origin of TAMs in lung cancer [143]. For example, the group 1 cluster was mainly composed of alveolar macrophages, which are pulmonary RTMs. On the other hand, the group II cluster comprised mDMs, which do not differentiate into RTMs, including at late stages of NSCLC. Moreover, RTMs and mDMs cohabit the TME throughout tumor growth (equilibrium and escape processes of cancer immunoediting), giving rise to TAMs. Furthermore, accumulation of anti-inflammatory macrophages in the NSCLC TME suppresses NK cell cytotoxicity [147]. Moreover, immunologically cold NSCLC also harbors immunosuppressive TAMs, which overexpress genes involved in immunoglobulin (Ig)-mediated immune responses, which might be critical for Ab-dependent cancer cell phagocytosis (ADCP) that supports their immunosuppressive function. TAMs also overexpress genes associated with extracellular matrix organization, such as MMPs and collagen-associated genes, suggesting their enhanced matrix remodeling activity [148].

The higher accumulation/activity of M2-like TAMs in the TME core of the NSCLC supports their interaction with T cells via immunosuppressive ligand–receptor interactions [149]. For example, secreted phosphoprotein 1 (SPP1; osteopontin)-CD44 and NECTIN2 (Nectin cell adhesion molecule 2 or CD112/poliovirus receptor-related-2)-TIGIT (T cell immunoreceptor with Ig and ITIM domain) axes were identified as major immunosuppressive pathways, with SPP1 secreted by TAMs contributing to immune evasion [149]. Elevated HLA-E and CD8B interaction with M2-like TAMs and T cells suggests modulation of the Ag presentation mechanism, which might suppress the cytotoxic action of CD8^+^T cells. Moreover, HLA-E interaction with the CD94/NKG2A receptor (an inhibitory NK cell receptor) of NK cells suppresses their cytotoxic function and helps cancer cells escape recognition and elimination [150,151,152]. Furthermore, elevated SPP1 or osteopontin induces PDCD1 (programmed cell death 1 protein) and CD160 overexpression, which further confirms the development of an immunosuppressive TME or cold tumor immunophenotype [149], whereas TMEs of patients with NSCLC, along with M2-like immunosuppressive TAMs having a strong/hot M1-like signature (M1^hot^), exhibit a high density of CD8^+^ tissue-resident memory T cells (T_RMs_) [153]. The higher accumulation of M1^hot^ TAMs increases patient survival due to CXCL9-dependent increased recruitment of CD8^+^ antitumor T cells, including TRMs, which create a “hot” tumor immunophenotype, which can be effectively targeted with available cancer immunotherapies.

The existence of RTMs (alveolar macrophages) in the TME lesions of patients with NSCLC has been confirmed [143]. RTMs were decreased in the lungs of patients with NSCLC as compared to healthy controls, but mDMs increased with advancing cancer. These early tumor RTMs highly express major histocompatibility complex-II (MHC-II) and T cell chemoattractants (CCL9 and CCL17), along with peptidases (MMP12, MMP14, and a disintegrin and metalloproteinase domain-like decysin-1 or ADAMDEC1) and integrin-binding molecules (Tetraspanin 4 or TSPAN4). However, IL-1β, NLRP1B (NLR family, pyrin domain containing 1B), Wnt signaling molecules (Amer2), and cell adhesion and migration molecules were decreased in these RTMs [143]. Moreover, MHC-II-encoding genes stay open throughout the cancer progression. It is interesting to note that these RTMs minimally alter their epigenetic and transcriptional landscape in response to early tumor seeding, and most of the transcriptional changes are associated with Ag presentation and tissue remodeling [143]. Thus, early upregulation of MHC-II on RTMs during initial phases of NSCLC indicates their role in Ag presentation to helper CD4^+^T cells. However, the expression of VEGFA and urokinase-type plasminogen activator (uPA) by Kirsten rat sarcoma virus oncogene homologue (KRAS, a potent proto-oncogene)/p53 mutant cancer cells generates an immunosuppressive environment that supports differentiation of CD4^+^T cells to T_regs_ [143,154]. Notably, mDMs and RTMs are similarly efficient in transforming naïve CD4^+^T cells to T_regs_, but RTMs were uniquely able to increase the expression of CD73 and cytotoxic T lymphocyte-associated protein 4 (CTLA4, an immune checkpoint) on differentiated T_regs_ more than mDMs.

Patients with colorectal cancer (CRC) exhibiting higher microtubule-associated protein 1A/1B light chain 3 (LC3, a central player in autophagy and autophagosome formation) expression and activity also exhibit a higher M1^hot^ TAM accumulation [155,156]. High M1^hot^ TAM accumulation is correlated with tumor-infiltrating antitumor CD4^+^, CD8^+^, and CD20^+^ lymphocytes, inducing a “hot” tumor phenotype that responds well to anticancer immune and chemotherapies. Thus, autophagy in the TME also regulates macrophage polarization toward the anticancer M1 phenotype and the antitumor immune response. However, it must be kept in mind that autophagy in tumorigenesis depends on the tissue/organ and the autophagy gene, which might suppress or activate antitumor immunity depending on the cancer-type and involvement of the autophagy gene, as discussed in detail elsewhere [157,158].

Classically activated macrophages highly express MHC-II molecules, which support the generation of a Th1 immune response but not potent cytotoxic T cells. However, cluster analysis studies do not support a signature that discriminates between macrophage activation states as classical (M1, pro-inflammatory) versus alternative (M2 or anti-inflammatory or immunosuppressive macrophages) [159]. Moreover, further nomenclature and experimental guidelines by experts in macrophage biology have been issued to be followed when studying macrophages. For example, under in vitro experimental conditions, the source of macrophages and the external stimuli used must be mentioned, and the use of regulatory macrophages, as all macrophages are immunoregulatory in some capacity [160]. Tissue/organ macrophages [peritoneal macrophages, Kupffer cells (liver macrophages), microglia (brain macrophages), alveolar macrophages (present in pulmonary alveoli), and Langerhans cells (skin macrophages] exhibit functional heterogeneity, which might affect their immunological functions in target organ cancer pathogenesis and the immunoediting process [161,162,163]. Although major populations of serous cavity-resident mononuclear phagocytes among humans and mice share common features, the proportions of different macrophage differentiation stages differ greatly between the two species [164]. Moreover, DC2-like cells are more prominent in human peritoneal and serous cavity immune cell populations than in mice.

Now, we know that TAMs, comprising local/residential macrophages and mDMs, comprise up to 50% of the tumor mass [163,165]. The macrophage diversity (mDMs and RTMs, comprising TAMs) across different cancers has been discussed in detail elsewhere [166]. TAM accumulation in the TME is tumor-friendly and associated with poor prognosis in most human cancers [167,168]. A common macrophage signature in large datasets derived from over 1000 patients with tumors of several different origins has been demonstrated, which includes many known macrophage markers, CD68, CD163, CD14, and colony-stimulating factor 1 receptor (CSF1R), and many lysosomal genes, alongside MHC-II and costimulatory molecules (CD86), but does not fit with an M1 versus M2 paradigm [169]. This study indicates that the TAM signatures do not depend on tumor cell type. Moreover, MHC-II expression by TAMs is epigenetically controlled by the decoy receptor 3 (DcR3, a member of the TNF receptor superfamily upregulated in tumors) [170]. DcR3 overexpression in cancer cells decreases MHC-II expression by TAMs, which downregulates their Ag presentation capacity to activate the Th1 immune response. DcR3-mediated MHC-II expression downregulation in TAMs involves ERK- and JNK-induced deacetylation of histones associated with the class II transactivator (CIITA) promoters. Human cancers with upregulated DcR3 expression exhibit higher numbers of TAMs without HLA-DR expression [170]. However, recent single-cell RNA-sequencing data from 10 cancer types have indicated that TAMs with higher MHC-II expression (MHC-II^high^TAMs) interact and activate TME T_regs_ via Ag presentation and direct contact [171]. Moreover, TMEs of patients with cancer having a higher number of MHC-II^high^TAMs receiving immunotherapy show resistance to therapy. Thus, both up- and downregulation of MHC-II expression by TAMs in patients with cancer prove detrimental to them by supporting immunosuppression and resistance to immunotherapy. Further study of DcR3 expression in the TME and MHC-II expression by TAMs is critical to explore TAM-associated cancer immunoediting and to boost current immunotherapies depending on DcR3 expression.

Interestingly, a mouse (C57BL/6) model of pulmonary metastasis of Lewis lung carcinoma (LLC) injected subcutaneously has indicated that metastasized tumor mDMs have impaired expression of MHC-II molecules but overexpress prostaglandin E_2_ (PGE_2_)-inducible genes, such as arginase 1 (*Arg1*) [172]. These *Arg1*-expressing TAMs generate a T_reg_ subset called Th1-T_regs_ by secreting platelet factor 4 (PF4 or CXCL4) to support immunosuppressive TME in melanoma-bearing mice [173]. PF4/CXCL4 induces generation of Th1-T_regs_ subtypes of T_regs_ via interacting with its cognate receptor CXCR3. However, CXCR3 has several other ligands, including CXCL9, which also increases T_regs_ in the TME to support immunosuppression and decrease the antitumor CD8^+^T cell population [173,174]. In early stages of lung adenocarcinoma (LUAD), TAMs do not exhibit a specific pro- or anti-inflammatory signature; instead, they express similar levels of gene expression for pro- and anti-inflammatory activity and exhibit pro-tumorigenic function [175].

Moreover, pro-inflammatory cytokines, such as IL-1β released from these TAMs, induce overexpression of E74-like factor 3 (ELF3, an important member of the E-twenty-six (ETS) TF family), which triggers the PI3K/Akt/NF-κB signaling pathway and overexpression of proliferation and anti-apoptosis genes, like Bcl-2-like protein 1(BCL2L1) and cyclin D1 protein (CCND1) [175]. However, at early stages of LUAD, when elimination of cancer cells is critical, infiltration of immunosuppressive tumor-infiltrating myeloid (TIM) cell subsets, including monocytes, compromises antitumor NK and T cell-mediated immunity [154]. Further single-cell RNA sequencing studies from LUAD patient samples revealed the increased accumulation of mDMs with pro-inflammatory activity in the TME and its association with high histological grade and worse prognosis at later stages [176]. On the other hand, LUAD patients with higher non-inflammatory mDMs, NK cells, and conventional T cells show better prognosis, indicating that accumulation of pro-inflammatory mDMs, comprising the major TAM population at later stages (equilibrium stage of cancer immunoediting), is detrimental.

Further *in vitro* studies have indicated that PGE_2_ expression in mDMs prevents MHC-II expression but promotes arginase 1 expression via the PGE_2_ receptor called PGE_2_ receptor 2 (EP2), which is further accompanied by DNA methylation. Thus, PGE_2_-EP2 receptor interaction-derived DNA methylation is critical to determine the differentiation of infiltrated monocytes into mDMs and their MHC-II expression. Moreover, PGE_2_ also suppresses pro-inflammatory activity of macrophages via EP2- and EP4-mediated downstream cAMP-CREB (cAMP responsive element binding protein) signaling-mediated induction of Krupple-like factor 4 (KLF4) and induces IL-10 production, promoting M2-like immunosuppressive macrophages supporting tumorigenesis [177,178,179]. These TAMs also produce PGE_2_, which drives exhaustion of cytotoxic CD8^+^T cells and resistance of PD-1/PD-L1-based ICIs [180].

Moreover, EP2 receptor KO mice subjected to pulmonary metastasis exhibit decreased lung metastasis and increased presence of MHC-II^+^mDMs [172]. Thus, MHC-II expression by TAMs is a critical process in tumor immunopathogenesis and cancer immunoediting and must be explored efficiently in different human tumors to develop tumor- and macrophage-specific immunotherapy. Furthermore, human macrophages express MHC-II at a higher level than murine macrophages, which may impact their APC and T cell stimulatory action depending on the target organ cancer studied [13,181,182]. Moreover, MHC-II expression on murine gut macrophages supports T cell homeostasis and is governed by the gut microbiota [183]. The germ-free, antibiotic-treated, and MyD88^−/−^ mice show a significant decrease in MHC-II expression on their gut macrophages, suggesting gut-microbiota-driven mechanisms. This gut-microbiota-dependent MHC-II expression on human macrophages (gut, lung, and skin) is not yet known and has the potential to explore microbiota-dependent macrophage-dependent cancer immunoediting in cancers of these organs. Therefore, species-specific differences between macrophages must be considered under experimental conditions by studying their role in different cancers and the associated immunoediting process.

Phagocytosis of cancer cell-derived debris by mDMs triggers a pro-tumorigenic program governed by triggering receptor expressed on myeloid cells 2 (TREM2) [184]. The TREM2-expressing macrophages decrease antitumor cytotoxic NK cell activity by modulating IL-18/IL-18BP decoy interactions and IL-15 production [184]. The depletion of TREM2 increases TME NK cell accumulation and NK cell-mediated killing of cancer cells in a lung cancer model [184]. TREM2 depletion also increases proinflammatory antitumor cDC1s, which are critical for cross-Ag presentation for inducing TCMI. TREM2^+^ macrophages have also been observed in biopsies of patients with NSCLC. Thus, tumors rich in mDMs and local macrophages expressing TREM2 due to efferocytosis of tumor cell-derived Ags show decreased NK cell infiltration, cDC1 activity, and antitumor immune response. The details of TREM2 in the TIME and TAMs and its inhibition to increase the efficacy of cancer immunotherapy have been discussed elsewhere [185,186,187]. It is important to mention that TREM2 expression in glioblastoma (GBM) is not associated with immunosuppression but shows a strong positive association with the canonical phagocytosis markers lysozyme (LYZ) and macrophage scavenger receptor (CD163) in gliomas [188]. TREM2 targeting must be dependent on tumor-type, where its expression is associated with the immunosuppressive TME/TIME. Moreover, microglia and mDMs are critical in the progression of pediatric high-grade gliomas [189]. *CCL8^−/−^* and *CCL12^−/−^* mice subjected to glioma show decreased myeloid cell infiltration along with increased antitumor lymphoid cells (T cells), leading to their increased survival. Pharmacological inhibition of CCR4 and CCR5 has also indicated similar findings with increased survival, indicating inhibiting microglial activity and mDM infiltration in the glioma can be an immune-modifying therapeutic approach. Furthermore, higher mDM infiltration in the glioma TIME is epigenetically governed; for example, diffuse midline gliomas (DMGs) with histone mutations, such as H3.3K27M, have higher infiltration of mDMs [189].

ADCP of cancer cells by macrophages decreases their anticancer activity. It upregulates their anti-inflammatory action, such as upregulation of pro-angiogenesis and immunosuppressive chemokine genes [190]. These macrophages exhibiting ADCP also show upregulated expression of cellular, oxidative, and lysosomal stress TFs. This gene regulatory program is shared by macrophages showing ADCP or phagocytosis of apoptotic cancer cells [190]. Thus, the epigenetic reprogramming among TAMs upon ADCP or phagocytosis of apoptotic cancer cells induces an anti-inflammatory, tumor-supportive, proangiogenic stage that helps in cancer growth, survival, and metastasis across different cancer types [190]. Moreover, phagocytosis of cancer cells by TAMs also induces their immunosuppressive action by promoting oxidative phosphorylation (OXPHOS), which is also true for tumor-supportive DCs in the TME [191]. Thus, cancer cell phagocytosis by TAMs and TME DCs alters their immunometabolic reprogramming, which supports their immunosuppressive action.

The efferocytosis (phagocytosis of apoptotic cells) of dying cancer cells of PDAC by macrophages supports metastasis of pancreatic cancer to the liver through reprogramming macrophages to exhibit immunosuppressive function [192]. Progranulin (a lysosomal immunoregulatory protein) is critical for the process of efferocytosis, and its deletion has protected against PDAC metastasis to the liver by restoring antitumor T cell activity [192,193]. Macrophage-derived Progranulin and cancer cell (PDAC)-derived leukemia inhibitory factor (LIF) induce myofibroblastic metastasis-associated fibroblasts (MAFs) or myMAFs via STAT3 signaling [194]. In return, myMAFs secrete osteopontin, which supports immunosuppressive macrophages, leading to the suppression of antitumor cytotoxic T cells. During PDAC liver metastasis, liver Kupffer cells exhibit a proinflammatory phenotype in response to metastatic cancer cells and induce fibrotic lesions in the liver [192]. In addition to dysregulation of local liver Kupffer cells, mDMs are also infiltrated into the liver and exhibit immunosuppressive function to support liver metastasis. Thus, under non-cancer conditions, macrophages clear apoptotic cells via efferocytosis to maintain homeostasis, but efferocytosis by TAMs supports the local immunosuppressive niche in the liver to support liver metastasis. Thus, in PDAC metastasis to the liver, both pro- and anti-inflammatory macrophages play critical roles. For example, necroptotic cell death of PDAC cells during early liver metastasis T-stage (T1M1) due to overexpression of mixed lineage kinase domain-like pseudo-kinase (MLKL) drives macrophage recruitment and activation, but also induces CD47 (a do-not-eat-me signal) expression by PDAC cells [195]. These macrophages secrete macrophage extracellular traps (METs) in response to CXCL8 release induced by MLKL-triggered PDAC cell necroptosis. CXCL8 also upregulates ICAM-1 expression on pancreatic cancer cells, promoting their interaction with endothelial cells to support their liver metastasis. Furthermore, METs create a prometastatic extracellular matrix (ECM) niche, which supports PDAC liver metastasis. Inhibition of MET formation and CD47-blocking Ab treatment suppressed the T1M1-PDAC metastasis [195]. It is important to note that MLKL-driven PDAC cell necroptosis occurs independently of RIPK3 (receptor-interacting serine/threonine-protein kinase 3).

TAMs of mammary tumors in mice are phenotypically and functionally different from local normal mammary tissue macrophages (MTMs) [196]. The accumulation of immunosuppressive TAMs in breast cancer is also associated with poor prognosis and clinical outcome, due to suppressing infiltration and activity of antitumor T and NK cells [197]. These TAMs express VCAM-1 and proliferate upon differentiation from inflammatory monocytes; their terminal differentiation depends on the Notch signaling TF, Recombination Signal Binding Protein for Immunoglobulin Kappa J Region (RBPJ), which is a Notch signaling inhibitor [196]. TAM depletion (mediated by tumor-infiltrating inflammatory monocytes) restores cytotoxic T cell infiltration and the antitumor immune response, and suppresses tumor growth [196]. The myeloid cell-specific RBPJ deletion increased the number of M2-like macrophages due to increased M1 to M2 polarization [198]. Notch signaling is a critical determinant of TAM differentiation into M1 (proinflammatory) versus M2 (anti-inflammatory or immunosuppressive) macrophages, as its absence/inhibition is associated with the development of M2-like TAMs [199]. Moreover, RBPJ is required for M2 macrophage polarization [200]. The RBPJ-mediated Notch signaling supports M1 versus M2 polarization through suppressor of cytokine signaling 3 (SOCS3) [199]. SOCS3 signaling regulates M2 macrophage polarization, improves the quality of the IL-6 response, and inhibits the IFN-induced gene expression program supporting M1 macrophage polarization [201,202]. Thus, SOCS3 deficiency in macrophages promotes M1 macrophage polarization and inflammation, whereas its activation supports M2 macrophage polarization, which promotes immunosuppression in the TME, supporting tumor growth and metastasis.

These TEK- or tunica intima endothelial kinase 2 (Tie2; angiopoietin-1 receptor, CD202B)-expressing TAMs are not present in nonneoplastic tissue of healthy individuals [113]. The Tie2 ligand (Angiopoietin-2) released by activated endothelial cells and angiogenic vessels of the TME attracts Tie2-expressing monocytes/macrophages (TEMs), serves as TAMs, and supports tumor angiogenesis along with immunosuppression [113,203]. These TEMs express highly proangiogenic genes, like MMP-9, VEGF-α, cyclooxygenase-2 (COX-2), and wingless-related protein 5A Wnt5A). However, the Tie2–Angiopoietin-2 interaction further increases the angiogenic potential of TEMs by upregulating two proangiogenic enzymes, thymidine phosphorylase (TP) and cathepsin B (CTSB), and M2 markers, such as IL-10, mannose receptor (MRC1), and CCL17 [203]. Thus, TAMs support cancer-associated angiogenesis and promote an immunosuppressive TME (TIME) for cancer growth and survival—proliferation and metastasis.

The tumor-initiating cells (TICs) utilize TAMs to evade their immunorecognition and clearance (Figure 3) [204]. For example, TIC-derived growth arrest-specific 6 (GAS6) interacts with AXL receptor tyrosine kinase/MER proto-oncogene (AXL/MERTK), a tyrosine kinase of TAMs, called immunoregulatory TAMs (Reg-TAMs), that create and support immunosuppressive TME by releasing CCL8 that promotes cancer cell stemness and invasion, along with recruiting T_regs_ by interacting with CCR1 and CCR5 [204,205,206]. Inhibition of the GAS6/AXL/MERTK axis not only reactivates antitumor immunity but also enhances the efficacy of ICIs. Ag presentation via TAMs drives T cells from a progenitor exhaustion state to terminal exhaustion, and their depletion increases the efficacy of ICIs and decreases T cell terminal exhaustion [207]. Furthermore, TAM depletion activates transcription factor 4 (TCF-4), which activates a fibrotic pathway leading to the development of collagen-III-rich networks in the TME, thereby supporting the infiltration of antitumor T cells and neutrophils [208]. Thus, targeting TAMs can be a novel anticancer therapeutic approach that will not only eliminate tumor-supportive immunosuppressive TAMs but also their neoangiogenic effects, which are critical for tumor survival and growth (Figure 4B) [167,209,210,211,212]. The details of TAMs, their clinical relevance, and their regulatory role in the TME as an innate immune cell population and in the orchestration of angiogenesis, extracellular matrix remodeling, cancer cell proliferation, metastasis, and immunosuppression, along with suppressive effects on cancer chemotherapies and ICIs, have been discussed elsewhere [167,213,214,215,216,217].

### 3.2. Development of Macrophage Targeting-Based Cancer Immunotherapies, Including Chimeric Antigen Receptor (CAR)-Macrophages (CAR-Ms)

Different macrophage-targeting strategies, such as inhibitors of cytokines and chemokines involved in the recruitment and polarization of tumor-promoting myeloid cells, and activators of their antitumorigenic and immunostimulatory functions, have been developed and discussed in detail elsewhere [167,168,218]. Moreover, understanding the important role of macrophages at all three stages of cancer immunoediting/3Es has further advanced macrophage-based solid cancer cellular immunotherapeutic approaches through developing human CAR-Ms able to address key limitations of CAR-T cell therapies, which proved beneficial in hematologic malignancies but faced different challenges in solid cancers [219,220,221,222,223]. These CAR-Ms have a greater capacity to penetrate the solid cancer TME, exhibit greater phagocytic activity against cancer cells, and maintain a sustained proinflammatory M1 phenotype and function. Moreover, these CAR-Ms reprogram bystander M2 macrophages to M1, upregulate Ag presentation machinery, recruit and present antigens to T cells, and resist the effects of immunosuppressive cytokines present in the TME [219,224]. The details of CAR-Ms in cancer immunotherapies, along with their development strategies and clinical trial stages, have been discussed elsewhere [225,226,227,228].

Three generations of CAR-Ms have been developed so far. For example, first-generation CAR-Ms are CD3-ζ-based and phagocytose cancer cells in an Ag-dependent manner. In contrast, second-generation CAR-Ms called CAR-iMs are engineered induced pluripotent stem cell (iPSC)-derived macrophages (iMs) with TLR4/intracellular toll/IL-1R (TIR) domain-containing CARs, exhibiting greater antitumor activity than first-generation CAR-Ms [226]. Thus, the discovery of TLR4 not only advanced fundamental immunology and the understanding of inflammatory processes/diseases but also improved CAR-M-based cancer immunotherapies. Moreover, CAR-iMs generated from human iPSC-derived macrophages targeting prostate stem cell antigen (PSCA), expressing membrane-bound IL-15 and truncated epidermal growth factor receptor (EGFR) for immune cell activation and a suicide switch, have shown strong antitumor activity against human pancreatic cancer (*in vitro* and *in vivo*) in experimental conditions [229]. These CAR-iMs have more potent antitumor activity than first-generation CAR-Ms and do not exhibit signs of cytokine release syndrome and other in vivo toxicities. Third-generation CAR-Ms have been developed by integrating nanobiotechnology and *in vivo* reprogramming [225]. For example, the generation of third-generation CAR-Ms involves nanocarriers that, along with *in vivo* reprogramming of macrophages, also deliver plasmid DNA encoding CAR-IFN-γ [230]. This CAR-IFN-γ enhances the M1-like phenotype and its proinflammatory, phagocytic, and antitumor activity. The continuous advances in macrophage biology and their role in tumor immunology have led to the development of this novel technology to fight solid cancers, with CAR-Ms in different stages of clinical trials, as mentioned elsewhere. CAR-Ms have some limitations; for example, like CAR-T and CAR-NK cells, they do not expand and survive long after infusion and require repeated infusions and adjunctive therapies to achieve therapeutic efficacy, which limits their long-term immunosurveillance in patients with cancer [225,229,231]. However, these limitations of CAR-Ms have been addressed by second- and third-generation CAR-Ms, along with continuous advances in the field. The details of CAR-Ms and their developmental stages (preclinical and clinical stages) have been mentioned elsewhere [225,232,233].

Moreover, CAR-Ms with built-in CD47 blockers have been developed to fight against tumor Ag heterogeneity and activate antitumor T cells via Ag cross-presentation [234]. These CAR-Ms express an enhanced synthetic phagocytosis receptor (eSPR), which integrates an FcRγ-driven phagocytic CAR with built-in secreted CD47 blockers. The engineered eSPR empowers CAR-Ms to combat tumor Ag heterogeneity and stimulate antitumor CD8^+^T cells via phagocytosis-dependent Ag cross-presentation [234]. CD47 on cancer cells recognizes SIRPa (signal regulatory protein-α) on macrophages and DCs to avoid phagocytosis; therefore, CAR-Ms with a humanized single-chain variable fragment with FcγRIIa and integrating short hairpin RNA to silence SIRPα have been developed to disrupt the CD47-SIRPa-mediated do-not-eat-me signaling pathway [235]. These CAR-shSIRPa-M cells increase antitumor cytotoxic TCMI to fight against solid cancers. SIRPa inhibition in CAR-shSIRPa-M cells increases their proinflammatory potential. It increases the cGAS/STING signaling pathway to generate type 1 IFNs and NF-κB-dependent proinflammatory molecules to exert an antitumor immune response [235].

Interestingly, in colorectal cancer, SIRPa-expressing TAMs and DCs exert pro-tumorigenic effects independent of their interaction with CD47 expressed on cancer cells [236]. For example, SIRPα^−/−^ macrophages exhibit increased phagocytosis of colorectal cancer cells and Ag presentation to enhance TCMI by mediating CCL8-dependent T cell recruitment. Thus, use of phagocytosis checkpoint inhibitor-based therapies must be used depending on cancer type, and further studies are needed in this area. Moreover, administration of anti-CD47 Abs with adoptively transferred T cells decreases the efficacy of T cell-based therapy due to clearance of CAR-T cells by macrophages. Therefore, CAR-T cells expressing the CD47 variant CD47(Q31P) (47_E_) have been developed, which avoid their phagocytosis by recognizing SIRPa and can be used with anti-CD47 Abs, mediating substantial macrophage recruitment to the TME [237]. This approach has utilized T cell-based and macrophage-mediated antitumor immune responses while fighting against solid cancers. Moreover, dual phagocytosis checkpoint inhibitor strategies, utilizing CD24 and CD47 blockers, have been developed, which increase the phagocytic potential of macrophages, their type 1 IFN secretion potential, and Ag cross-presentation [238]. Dual phagocytosis checkpoint inhibitor strategies, utilizing CD24 and CD47 blockers, further increase the efficacy of PD-1 blockers (ICIs), as indicated by increased survival of mice with GBM. Hence, phagocytosis checkpoint blockers have the potential to increase the efficacy of ICIs and need further preclinical and clinical studies. Thus, understanding the importance of macrophages during carcinogenesis has established their role in cancer immunosurveillance and immunoediting and led to the development of different strategies targeting macrophages, including TAMs to fight against cancer.

## 4. Emergence of Ag-Presenting Cells (APCs) and Discovery of DCs Led to Development of DC-Based Anticancer Vaccines

**Figure 5:**
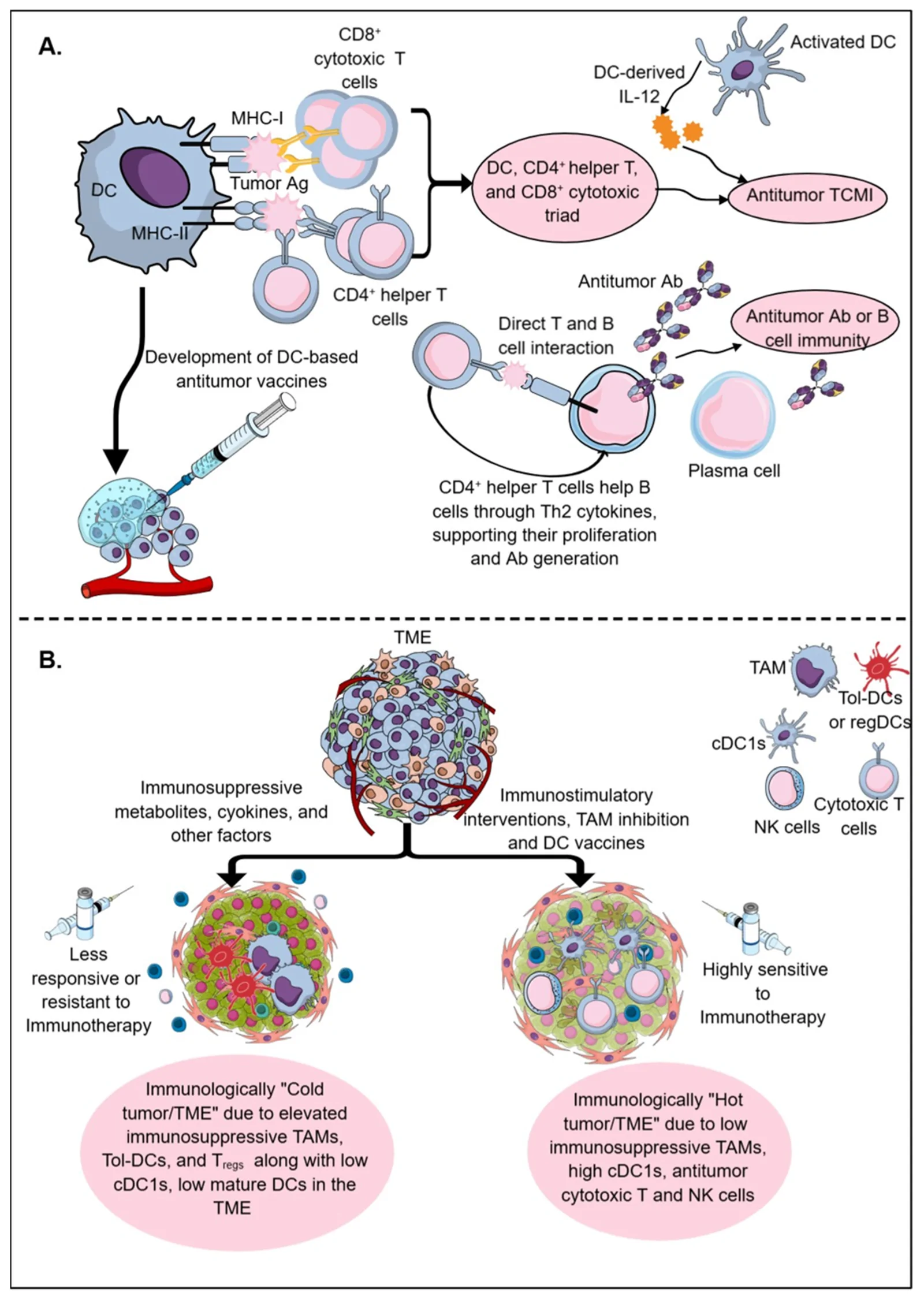
DCs in tumor immunity and immunoediting. (**A**). DCs in tumor immunity. DCs are potent APCs with steady MHC-I and -II expression. MHC-I and -II are critical for Ag, including tumor Ag presentation to CD8^+^ cytotoxic and CD4^+^ helper T cells to exert potent anticancer/antitumor TCMI. The activated CD4^+^ helper T cells further influence B cells by direct interaction and producing Th2 cytokines (IL-4, 5, and 6). This helps in the proliferation and maturation of B cells into Ab producing plasma cells. Moreover, activated DC-derived IL-21 exerts antitumor immunity by supporting TCMI, which is critical for elimination phase of cancer immunoediting. DC discovery and recognition of their immunological functions have led to the development of different DC-based cancer vaccines. (**B**). TME affects DC maturation and Ag presentation processes causing dysregulation of DC-mediated antitumor T and NK cell immune response. The immunosuppressive metabolites, cytokines and other factors released by cancer cells and TAMs suppress DC maturation and lead to development of Tol-DCs or regDCs with immunosuppressive functions, suppressing antitumor TCMI and NK cell activity. This creates an “immunologically cold” tumor/TME. This tumor type shows low efficacy to available immunotherapies, whereas tumors/TMEs with high pro-inflammatory/stimulatory DCs, such as cDC1s, cytotoxic T and NK cells, and low TAMs are “immunologically hot”. These tumors are highly responsive to available cancer immunotherapies, including ICIs. Details are mentioned in the text.

Simultaneously, when cancer immunosurveillance was proposed in 1970, two major innate immune discoveries/concepts emerged: (1) emergence of the APC concept by Mishell and Dutton (Scripps Research Institute) in 1966, which was further supported by Mosier in 1967 by the finding that two different cell types are required for antibody (Ab) generation, and (2) discovery of potent innate immune cells serving as robust APCs, called DCs by Ralph Steinman and Zanvil Cohen at Rockefeller University in 1973 [239,240,241,242]. So, during the evolutionary period of cancer immunosurveillance, macrophages and DCs had emerged in immunology, but their role in the process was yet to be explored. Later (in 1978 and 1979), Steinman et al. further showed DCs as potent APCs due to the expression of MHC molecules, such as MHC-I and -II, critically required to present Ags to cytotoxic and helper T (Th) cells for their immunological functions, including antitumor immunity (Figure 5A) [243,244]. For example, Steinman and Witmer showed that DCs are more (100–300 times) potent immune cells in inducing a mixed lymphocyte reaction (MLR, a well-known technique used to mimic T cell-mediated rejection of donor tissue during transplantation) than unfractionated spleen cells [244,245,246]. Steinman and other researchers showed that DCs have equal potency to stimulate cytotoxic TCMI and thymus-dependent Ab responses [247,248,249,250]. Moreover, the Steinman group and others later showed that DCs are more potent APCs than monocytes/macrophages to stimulate MLR or TCMI [251,252]. It must be kept in mind that the DC subsets (conventional or classical DCs (cDC1 and cDC2), plasmacytoid DCs (pDCs), monocyte-derived DCs or mo-DCs, and Langerhans cells) and their location are critical for T cell programming and vaccine design, as discussed in detail elsewhere [253].

The recognition that DCs are critical for TCMI and thymus-dependent Ab generation, serving as potent APCs, led to the development of DC-based cancer vaccines (Figure 2 and Figure 5A). Moreover, trials of DC-based vaccines in humans began within 25 years of their discovery and development, with little understanding of their role in the cancer immunoediting process, including their trafficking, maturation, and immune functions in Ag presentation, and maturation [254,255,256]. For example, Ralph Steinman (2003) ran the phase 1 clinical trial for a DC-based vaccine (single dose) in patients with stage IV melanoma, and he reported increased patient survival in responding patients without the involvement of melanoma-specific cytotoxic TCMI [257]. However, before Steinman’s group, Nestle et al. (1998) had used DCs (generated in the presence of granulocyte/macrophage-colony stimulating factor (GM-CSF) and IL-4, pulsed with tumor lysate or a cocktail of peptides capable of activating cytotoxic T cells, depending on the patient’s HLA haplotype) in patients with advanced melanoma [258].

Similarly, an approach with monocyte-derived DCs (moDCs) pulsed with Mage-3A1 (human melanoma antigen-3A1) tumor peptide in patients with advanced stage IV melanoma was applied later in 1999, and these patients received five DC vaccinations at 14-day intervals, indicating frequent expansion of cytotoxic T cells in patients showing tumor regression and metastasis resolution [259]. Interestingly, these researchers showed that intradermal DC vaccination had immune-boosting effects, as indicated by increased cytotoxic TCMI, whereas intravenous DC vaccine administration reduced the antitumor immune response [259]. These findings indicate that autologous DCs are safer as vaccine candidates, with no physical signs of autoimmunity, but have induced delayed-type hypersensitivity (DTH) in 11 out of 16 patients [258]. These initial findings even led Ralph Steinman to have a DC-based vaccine derived from his own DCs upon his diagnosis with pancreatic cancer back in 2007, which increased his survival for years. However, he died on 30 September 2011, 3 days before the announcement of the Nobel Prize winner in physiology or medicine [260].

### DCs in TME, Their Role in Cancer Immunoediting, and Improved DC-Based Vaccines/Strategies

It is interesting to mention that, like other immune cells, DCs within the TME/TIME undergo maturation and immune reprogramming in response to metabolites and factors secreted by growing cancer cells and local/migrated immune cells. These metabolites and factors (IL-10, TGF-β, and VEGF) suppress DC maturation, Ag presentation, and migratory capacity (Figure 5B) [129,261,262,263]. The details of DC maturation in cancer have been discussed elsewhere [262]. The suppressed antitumor DC activity conceals TCMI, leading to defective cancer immune surveillance or immune editing, such as a failed elimination phase, progression to an equilibrium and escape phase for tumor progression and metastasis, along with the development of resistance to tumor immunotherapies due to “cold tumor/TME” (Figure 5B). For example, a defect in DCs during cancer pathogenesis suppresses the formation of a cellular triad comprising CD4^+^ and CD8^+^T cells, which interact with the same DC, and are critical to target a cancer cell by the CD8^+^T cell [264]. Failure of triad formation, where CD4^+^ and CD8^+^T cells interact with the same DC in the intratumoral region, supports tumor progression despite the presence of equal numbers of CD4^+^ and CD8^+^T cells [264]. Moreover, T cell-based immunotherapies and ICIs fail in patients with cancer having lower/defective DCs (unable to produce IL-12) in their TME/TIME (Figure 5B) [264,265].

DC-derived IL-12 supports antitumor TCMI (Figure 4); therefore, the success of ICI therapy depends on DC-derived IL-12 and IFN-γ, along with T cell: DC crosstalk [265]. It is interesting to note that stromal remodeling, or peritumoral stroma mimicking embryonic provisional matrix remodeling, regulates cDC1 abundance and activity in the TME, which can impact immune therapies aimed at activating antitumor TCMI [266]. Moreover, cross-presentation of neoantigens (released from dead cells) by cDC1s to CD8^+^T cells can determine the immune visibility of the tumor mutational landscape and sculpt cancer evolution through immunoediting [267]. The TME glutamine level and its uptake by cDC1s are critical for cDC1-mediated induction of antitumor CD8^+^ cytotoxic TCMI and enhancement of their effector function as indicated by IFN-γ, TNF-α, and granzyme B (GzmB) expression [268]. For example, cancer cells and cDC1s compete for glutamine via sodium-coupled neutral amino acid symporter 2 (SNAT2) encoded by the SLC38A2 (solute carrier 38A2) gene, and its deficiency in cDC1s eliminates the antitumor effects of glutamine supplementation. The expression of SLC38A2 is higher in DCs than in macrophages and T cells [268]. However, cancer cells overexpress SLC38A2 more than DCs. The SLC38A2-dependent glutamine uptake by cDC1s and regulation of dependent CD8^+^T cell effector function in the TME requires folliculin (FLCN), which is redundant for cDC2s. FLCN is critical to TFEB (a nutrient- and stress-specific TF) activity and lysosomal function in cDC1s.

The FLCN deletion in cDC1s increases TFEB nuclear localization and lysosomal mass, which dysregulates endolysosomal homeostasis, leading to defective Ag presentation and T cell priming [268,269]. It is important to mention that glutamine supplementation exerts no effect on naïve CD8^+^T cell priming in tumor-draining LNs. The direct contact between human cDC1s and CD4^+^T cells increases their potential to increase cytotoxic TCMI against tumor cells by supporting Ag cross-presentation and T cell priming [270]. The activated CD4^+^T cells in response to cytosolic GMP-AMP synthase/stimulator of IFN gene (cGAS/STING) signaling also produce type 1 IFNs, which promote MHC-1 expression on cDC1s and thereby improve their capacity to activate cytotoxic TCMI [271]. Moreover, helped/licensed cDC1s via CD40 signaling also support initial CD4^+^T cell activation along with CD8^+^T cell priming, increasing the expression of co-stimulatory ligands for CD8^+^T cells and their survival (as indicated by increased *Bcl2l1* expression) during priming of antitumor immune response [272,273,274]. Thus, helped/licensed cDC1s existing in the TMEs of many tumor types increase infiltration of antitumor cytotoxic and helper T cells, overall patient survival, and efficacy of ICIs. Additionally, an active cDC1-NK cell axis in the TME is associated with increased overall survival and increased efficacy of PD-1 inhibitors in patients with metastatic melanoma [275,276]. The details of DCs and cDC1s in regulating TCMI in cancer immunity have been discussed extensively elsewhere [277].

Additionally, the stimulatory DC (SDC)-NK cell axis is critical to determine the efficacy of immune-checkpoint inhibitors (ICIs) targeting T cells for their antitumor activity [278]. Moreover, NK cells in the TME are critical to chemoattract SDCs by secreting a cytokine called Fms-related receptor tyrosine kinase 3 ligand (FLT3LG). FLT3LG is critical for B and DC development in mice and humans [279]. Notably, FLT3LG is critical in humans for monocyte development and in mice for NK cell development. NK cells, by producing cDC1 chemoattractants such as CCL5 and XCL1 attract cDC1s in the TME, which exert antitumor immunity by activating cytotoxic T cells [280]. In human cancers, intratumoral C-C motif chemokine ligand 5 (CCL5 or RANTES/Regulated on Activation Normal T cells Expressed Secreted), X-C motif chemokine ligand 1 (XCL1), and XCL2 expression correlate well with genetic signatures of NK cells and cDC1, which associates with increased overall patient survival. Moreover, PGE_2_ in the TME impairs NK cell activity and viability, including CCL5 and XCL1 production, by disturbing the NK cell-cDC1 antitumor axis [280]. TME PGE_2_ via cAMP signaling downstream of the PGE_2_-receptors EP2 and EP4 expressed on cDC1s induces their dysfunction due to loss of IRF8 and conceals their potential to activate anticancer CD8^+^ cytotoxic T cells [281].

It is important to mention that despite their good tolerance and safety profile, the clear therapeutic efficacy of DC vaccines has only been observed in less than 15% of patients with cancer [282]. The protective action of DC vaccines in patients with cancer involves activation and enhancement of local and systemic antitumor immune response [282]. DC vaccines conserve their antitumor action (enhancing local and systemic antitumor immunity) when administered along with chemotherapy in patients with cancer, such as triple-negative breast cancer (TNBC) [283,284]. Moreover, prostate cancer is the first cancer type to which a DC vaccine called Sipuleucel-T (Provenge^®^, comprising recombinant Ag, which must be incubated with autologous or patient-derived APCs) was approved by the USFDA for clinical use and increased the overall survival among patients with metastatic castration-resistant prostate cancer [282,285,286,287].

Currently, DC vaccines are at different preclinical and clinical stages of development for different cancers, which are discussed in detail elsewhere [277,282,288,289,290,291,292,293,294,295,296,297,298]. Moreover, immunomodulatory approaches combined with ICIs have been developed to induce the accumulation of activated cDC2s carrying tumor Ags in the tumor-draining LNs, which, via Ag cross-presentation to CD4^+^T cells, induce IFN-γ production and associated antitumor immunity in PDAC [299]. PDACs are one of the most aggressive human malignancies, exhibit resistance to current immunotherapies, and lack antitumor CD8^+^T cells [299,300].

Recently, an advanced *in vivo* approach has been developed to reprogram cancer cells to develop into DCs delivering transcription factors, such as PU.1 (Purine rich box-1 is encoded by *SPI1 (Spi-1 protooncogene),* an ETS-family transcription factor, regulating myeloid, DC and B cell functional programs), IRF8, and BATF3 (Basic Leucine Zipper ATF-Like Transcription Factor 3, controls cDC1 development), with an adenovirus vector [301]. This in vivo therapeutic approach has revolutionized DC vaccines for cancer, where cancer cells can be transformed to cDC1s, which present tumor Ags to local CD4^+^ and CD8^+^T cells and induce cancer cell elimination, a critical step in cancer immunoediting to clear cancer cells. Furthermore, transformation of cancer cells to cDC1s in response to infusion of an adenoviral vector loaded with PU.1, IRF8, and BATF3 led to recruitment and expansion of polyclonal cytotoxic and memory T cells in the TME/TIME along with the formation of the tertiary lymphoid structure (TLS) containing T, B, and stromal cells. This process led to the conversion of an immunologically cold tumor to a hot one, which shows better efficacy to tumor immunotherapies, including ICIs. This approach of converting local cancer cells to cDC1s promotes local and systemic antitumor immunity. It protects against metastasis, which can be further utilized to increase the efficacy of ICIs in a melanoma model. Further studies in this direction will provide DC-based approaches to target cancer.

The discovery of DCs, their role as potent APCs, and their further nomenclature and classification, including the production of tolerogenic DCs (Tol-DCs), has not only advanced fundamental immunology but also tumor immunology and the cancer immunoediting process [302,303]. For example, the production and increase of Tol-DCs, called mature regulatory DCs (mregDCs), in the TME/TIME, further supports the immunosuppressive TIME (Figure 4), helping tumor growth, survival, and metastasis. These Tol-DCs/mregDCs are produced in the TIME through different mechanisms, such as metabolites (e.g., lactate) (Figure 5) and/or extracellular vesicles (EVs) produced by cancer cells [304,305]. Tumor-derived lactate induces transformation of cDCs of the TIME to mregDCs by activating sterol regulatory element-binding protein 2 (SREBP2), a master regulator of the mevalonate biosynthetic pathway. The DC-specific suppression/inhibition of SREBP2 in the melanoma TME suppresses melanoma progression and activates CD8^+^T cell-specific TCMI [304].

These mregDCs are CD63^+^ mature DCs, which migrate to tumor-draining LN tissue and suppress DC Ag cross-presentation in trans but enhance tumor-promoting Th2 and T_reg_ differentiation. Along with the LNs of several animal/preclinical models of different cancers, mregDCs are also sentinel to the LNs of patients with melanoma [304]. On the other hand, tumor-derived EVs induce mregDC production by triggering the unfolded protein response (UPR) via the Protein Kinase R-like Endoplasmic Reticulum Kinase (PERK)-activating transcription factor 4 (ATF4) and Inositol-requiring enzyme 1 a (IRE1α- X-box binding protein-1 (XBP1) axis, which via SREBP2 and PPAR-a transcription factors (TFs), induce fatty acid oxidation (FAO) in TME DCs, suppressing their Ag-presenting capacity to T cells [305]. FAO in DCs increases protoporphyrin IX synthesis and indoleamine-2,3-dioxygenase 1 (IDO1) activity that drives kynurenine production and T_reg_ differentiation [306]. Moreover, FAO in DCs suppresses IL-6 and IL-12 production. The extracellular kynurenine is taken up by CD4^+^T cells via large neutral amino acid transporter-1 (LAT-1, encoded by *slc7a5, solute carrier family 7 member 5*), where it binds to cytosolic aryl hydrocarbon receptor (AhR) and induces its mobilization to the nucleus, where it supports upregulation of FoxP3 expression and its polarization to Tregs, and inhibits Th17 cell generation by suppressing retinoic acid receptor-related orphan receptor-γt (RORγt) expression [307].

MHC-II^+^CD11b^+^CD11c^+++^ DCs also acquire the phenotype of Tol-DCs or regulatory DCs (regDCs) [308]. These MHC-II^+^CD11b^+^CD11c^+++^ DCs (regDCs) inhibit cytotoxic antitumor CD8^+^T cells and suppress IFN-γ production and anergy of naïve T cells. These regDCs exhibit higher L-arginine uptake and its metabolism by arginase. The L-arginine deficiency in the TME decreases CD3ζ chain (encoded by the CD247 gene) expression on T cells, which is a critical component of the T cell receptor (TCR) complex, and thus its impaired expression and activity induce T cell anergy upon antigenic stimulation in the TME of different cancers [308,309]. In addition to decreased CD3ζ chain expression, T cells under limited L-arginine conditions also show diminished IFN-γ and IL-5 production, but not IL-2 production [310]. The GM-CSF secreted from tumor cells also induces generation of monocyte-derived immunosuppressive (CD11b^+^CD11c^+^MHC-II^+^CD24^+^CD64^low^F4/80^low^) DCs in a murine model of metastatic pancreatic cancer [311]. These immunosuppressive DCs uniquely express MGL-2 (macrophage galactose N-acetyl-galactosamine specific lectin 2 or CD301b) and PD-L2 (or PDCD1LG2 or programmed cell death 1 ligand-2/CD273) in the metastatic environment and induce T_reg_ expansion. These immunosuppressive DCs accumulate at secondary sites and promote cancer metastasis by creating an immunosuppressive niche comprising accumulation of T_regs_ and decrease in antitumor CD8^+^T cell activity.

Furthermore, cancer cells also induce conversion of immature myeloid DCs to secrete TGF-β, which are present in tumor-draining LNs and support T_reg_ proliferation, and thus the immunosuppressive TIME [312]. The immature myeloid DC-derived T_reg_ proliferation is MHC-II restricted. Thus, Ag presentation by MHC-II^+^DCs in the TME, along with suppression of cytotoxic activity of antitumor CD8^+^T cells, also promotes expansion of T_regs_, contributing to tumor-induced tolerance. Tumor-derived molecules, such as VEGF, IL-10, and IL-6, block DC differentiation via Janus-activated kinase 2/signal transducer and activator of transcription 3 (JAK2/STAT3) pathway activation and by inactivating NF-κB signaling, which further supports their tumor-promoting anticancer activity [313].

IL-12 is critical for effector cytotoxic activity of CD8^+^T cells, and its lower levels in the TME suppresses anticancer cytotoxic action of CD8^+^T cells, including CD8^+^ effector memory T (CD8^+^T_EM_) cells [314,315]. Moreover, IL-12 supports differentiation of CD4^+^T cells to CD4^+^Th1 cells, which support antitumor immunity by releasing TNF-α, IL-2, and IFN-γ [316,317]. IL-12 is also critical for expansion of cytotoxic NK cells by sustaining their survival [318]. The success of IL-12 antitumor immunotherapy requires NK cell-derived CCL5 (a DC chemoattractant), which enhances functional antitumor DC-CD8^+^T cell synapse formation in the TME for generating effective antitumor TCMI [319]. On the other hand, IL-6 is also critical for maintenance of the T cell compartment (including naïve and memory T cell proliferation) by inducing survival factors, such as Mcl-1, and differentiation of naïve helper T (Th) cells to Th17 cells [317,320]. IL-6 also cooperates with IL-1β to block the immunosuppressive activity of T_regs_ [320]. Moreover, IL-6 trans-signaling has been shown for the rapid induction of granzyme-B (GzmB) in CD8^+^T cells, which can be utilized for rapid generation of effector CD8^+^T cells for effective immunotherapies [321]. For example, in the liver, naïve T cell stimulation does not require innate immune stimulation and occurs in the absence of conventional co-stimulatory signals [321]. Thus, suppression of IL-6 and IL-12 release by Tol-DCs further suppresses the antitumor helper and cytotoxic TCMI and NK cell-mediated cytotoxicity.

Inositol-requiring-enzyme 1 alpha (IRE1α) acts as an endoribonuclease to process the mRNA of X-box binding protein-1 (XBP-1) TF to generate active spliced isoforms of XBP1 (XBP1s) [322]. DC-specific PPAR-a targeting reversed their pro-tolerogenic activity in response to tumor-derived EVs. Moreover, addition of a PPAR-α inhibitor overcomes resistance to anti-PD-1-based ICIs and increases CD8^+^T cell-mediated antitumor immunity [305]. These Tol-DCs secrete IL-10 and TGF-β, which suppress antitumor TCMI and the pro-inflammatory innate immune response, while supporting the generation of immunosuppressive T_regs_ in the TME/TIME [302]. Moreover, these Tol-DCs have been utilized as a prognostic biomarker for tumors, such as Wilm’s tumor [323]. The development of Tol-DCs in the TME and their role in creating and supporting the immunosuppressive TIME, critical for tumor evasion (Figure 5), have been discussed elsewhere in detail [324,325]. Thus, Tol-DCs are critical players of equilibrium and evasion/escape phases of cancer immunoediting.

A recent study has indicated that common DC progenitor-derived pDCs (CDP-pDCs) with captured tumor Ags migrate to the thymus in response to CCR9-CCL25 interaction. In the thymus, these CDP-pDCs, via Ag presentation, induce clonal deletion of tumor-specific T cells [326]. Thus, tumor progression inhibits further antitumor T cell generation by promoting common lymphoid progenitor (CLP)-derived pDCs (CLP-pDCs) within the thymus. These CLP-pDCs further produce type 1 IFNs and alter thymus function [326]. This indicates that tumor-derived Ags captured by CDP-pDCs and the generation of CLP-pDCs within the thymus induce central tolerance against peripheral Ags, which helps in the induction of systemic immunosuppression and tumor metastasis. Tumor progression is associated with thymus reduction/shrinkage, indicating impaired T cell development and associated immune response [326].

Hence, the discovery of DCs has filled the gap in innate immunity-based regulation of adaptive immunity in the process of cancer immunoediting, along with providing the development of DC-based cancer vaccines and immunomodulatory approaches to enhance the efficacy of available cancer immunotherapies. Furthermore, the discovery of DCs has increased our understanding of the TME/TIME, along with local and distant DC-mediated immunosuppression critical for tumor growth, survival, proliferation, and metastasis, which was lacking at the time of the introduction of cancer immunosurveillance and immunoediting.

## 5. Conclusions

The concept of the TME was established before the introduction of cancer immunosurveillance, which later led to the development of cancer immunoediting. Now, depending on the accumulation and infiltration of anti- and/or pro-cancer immune cells, cancers are categorized as hot or cold (Figure 5B) [327]. The current classification of tumor immune phenotype (cold and hot tumors) mainly considers T cell enrichment and their pro- and antitumor immunity [328]. However, now we know that TAMs comprise 50% of the tumor mass and most tumor types. For example, tumors like skin cutaneous melanoma, bladder urothelial carcinoma, brain lower-grade glioma, cervical squamous cell carcinoma and endocervical adenocarcinoma, kidney renal papillary cell carcinoma, PDAC, thymoma, and sarcoma, behaving as cold tumors, have a higher TAM population with M2-like immunosuppressive properties along with lower cytotoxic T and NK cells (Figure 5B) [329]. This indicates that TAMs are critical players in determining tumor immunophenotype (cold and hot tumors) by regulating tumor-associated immune response depending on the target organ. Notably, the Ag presentation of both critical APCs, macrophages and DCs, gets dysregulated at all three stages of cancer immunoediting, depending on the tumor type. The dysregulated functions of TAMs and DCs critically determine the anticancer functions of NK and T cells (Figure 5B), on which the 2001 immunoediting concept was founded. Hence, exploring DCs and macrophages in cancer pathogenesis has provided critical information about carcinogenesis and cancer immunoediting, which was flawed at the time of the introduction of cancer immunoediting by Dr. Schreiber.

Now, DCs and macrophage-based cancer immunotherapies (vaccines) have been combined to increase the efficacy of CAR-T cell therapies against different solid cancers [330,331]. Moreover, phagocytosis checkpoints or do-not-eat-me signals [CD47, CD24, MHC-I, PD-L1, stannicalcin-1 (STC-1), and GD2 (G = ganglioside, D = sialic acid, and 2 = its order of migration on thin layer chromatography/TLC)], on cancer cells that prevent macrophage- and DC-mediated phagocytosis and the generation of a potent immune response, critical for their elimination, have been recognized as targets for cancer immunotherapies [332,333]. For example, pro-phagocytosis receptors, including Fc receptors, macrophage-1 antigen (MAC-1 or CD11b/CD18), and signaling lymphocytic activation molecule family member 7 (SLAMF7), expressed on macrophages, facilitate cancer cell elimination, and Fc receptors also increase the efficacy of several antitumor monoclonal antibodies in the clinic [333]. Anti-CD47 CAR-Ms have been developed to fight against different cancers, such as ovarian cancer, which further show enhanced efficacy to clear cancer cells in the presence of IL-21, which increases survival and antitumor action of CD8^+^T cells [334,335]. However, IL-21 use has a strong association with promoting a range of autoimmune diseases; therefore, its use needs caution [335]. To overcome IL-21-mediated autoimmune events, an IL-21 variant has been developed, which can be used with anti-CD47 CAR-Ms [336]. Further studies are needed in this direction.

Moreover, the trained immunity or innate immune memory concept was first proposed by Mihai Netea in 2011 in the context of infectious diseases [337]. According to trained immunity, innate immune cells (macrophages, DCs, and NK cells) remember previous immune stimuli, such as pathogenic infection, and exhibit robust innate immune responses based on this memory; epigenetic and metabolic reprogramming, without involving adaptive immunity, prevents reinfection [9,337,338]. Recently, a trained immunity-inducing nanobiologic called muramyl tripeptide_10_-high-density lipoprotein (MTP_10_-HDL) was developed, enhancing the efficacy of ICIs in an experimental melanoma model [339]. Moreover, intravenous MTP_10_-HDL treatment decreased the tumor size in the melanoma mouse model. It induced the expression of innate immune genes in hematopoietic stem cells (HSCs) and multipotent progenitors (MPPs) via epigenetic and metabolic reprogramming (two major pillars of trained immunity), leading to increased production of myeloid innate immune cells but a reduction in TAMs [339]. The TAM reduction is likely one of the mechanisms underlying the antitumor activity of MTP_10_-HDL and the increased efficacy of ICI. Thus, along with direct DC and macrophage targeting, their innate immune memory properties are being used to develop effective anticancer immunotherapies. Hence, innate immune memory in patients with cancer must be studied as it has the potential to determine their susceptibility/resistance to future cancer, as innate immune cells are the first line of defense and critical in the process of cancer immunoediting.

*In conclusion*, exploring and understanding the role of DCs and macrophages in carcinogenesis and associated immune dysfunction have filled immunological gaps that existed at the time of introduction of cancer immune surveillance and immunoediting. Understanding DC- and macrophage-based immunological events, such as phagocytosis, Ag presentation, development of immunosuppressive TAMs and Tol-DC/reg-DCs, MHC expression dysregulation, generation of tumor-supportive molecules, and their critical role in controlling NK- and T cell-based immune responses, has led to the development of DC- and macrophage-based immunotherapies. These macrophage- and DC-based immune therapies can be used alone or in conjunction with existing cancer therapies, including ICIs, which would not be possible without exploring their role in cancer immunopathogenesis, immunosurveillance, and immunoediting. Innate immune memory is an emerging area and must be studied in the context of cancer immunoediting.

## Data Availability

No new data were created or analyzed in this study. Data sharing is not applicable to this article.

## References

[1] Li J., Xiao C., Li C., He J. (2025). Tissue-resident immune cells: From defining characteristics to roles in diseases. Signal Transduct. Target. Ther..

[2] Rankin L.C., Artis D. (2018). Beyond Host Defense: Emerging Functions of the Immune System in Regulating Complex Tissue Physiology. Cell.

[3] Li J.H., Hepworth M.R., O’Sullivan T.E. (2023). Regulation of systemic metabolism by tissue-resident immune cell circuits. Immunity.

[4] Zhao J., Andreev I., Silva H.M. (2024). Resident tissue macrophages: Key coordinators of tissue homeostasis beyond immunity. Sci. Immunol..

[5] Masopust D., Soerens A.G. (2019). Tissue-Resident T Cells and Other Resident Leukocytes. Annu. Rev. Immunol..

[6] Gray J.I., Farber D.L. (2022). Tissue-Resident Immune Cells in Humans. Annu. Rev. Immunol..

[7] Allie S.R., Randall T.D. (2020). Resident Memory B Cells. Viral Immunol..

[8] Netea M.G., Joosten L.A., Latz E., Mills K.H., Natoli G., Stunnenberg H.G., O’Neill L.A., Xavier R.J. (2016). Trained immunity: A program of innate immune memory in health and disease. Science.

[9] Ochando J., Mulder W.J.M., Madsen J.C., Netea M.G., Duivenvoorden R. (2023). Trained immunity—Basic concepts and contributions to immunopathology. Nat. Rev. Nephrol..

[10] Liu K., Bradshaw R.A., Stahl P.D. (2016). Dendritic Cells. Encyclopedia of Cell Biology.

[11] Huang J.Y., Gerner M.Y. (2026). Dendritic Cell Organization and Function in Innate and Adaptive Immune Defense Within Lymph Nodes. Immunol. Rev..

[12] Huang J.Y., Gerner M.Y. (2026). Dendritic cells regulate the innate-adaptive balance in lymph nodes for optimal host defense. Cell.

[13] Guan F., Wang R., Yi Z., Luo P., Liu W., Xie Y., Liu Z., Xia Z., Zhang H., Cheng Q. (2025). Tissue macrophages: Origin, heterogenity, biological functions, diseases and therapeutic targets. Signal Transduct. Target. Ther..

[14] Lazarov T., Juarez-Carreño S., Cox N., Geissmann F. (2023). Physiology and diseases of tissue-resident macrophages. Nature.

[15] Mass E., Nimmerjahn F., Kierdorf K., Schlitzer A. (2023). Tissue-specific macrophages: How they develop and choreograph tissue biology. Nat. Rev. Immunol..

[16] Epelman S., Lavine K.J., Randolph G.J. (2014). Origin and Functions of Tissue Macrophages. Immunity.

[17] Gautier E.L., Shay T., Miller J., Greter M., Jakubzick C., Ivanov S., Helft J., Chow A., Elpek K.G., Gordonov S. (2012). Gene-expression profiles and transcriptional regulatory pathways that underlie the identity and diversity of mouse tissue macrophages. Nat. Immunol..

[18] Shankaran V., Ikeda H., Bruce A.T., White J.M., Swanson P.E., Old L.J., Schreiber R.D. (2001). IFNgamma and lymphocytes prevent primary tumour development and shape tumour immunogenicity. Nature.

[19] Dunn G.P., Bruce A.T., Ikeda H., Old L.J., Schreiber R.D. (2002). Cancer immunoediting: From immunosurveillance to tumor escape. Nat. Immunol..

[20] Dunn G.P., Old L.J., Schreiber R.D. (2004). The immunobiology of cancer immunosurveillance and immunoediting. Immunity.

[21] Schreiber R.D., Old L.J., Smyth M.J. (2011). Cancer immunoediting: Integrating immunity’s roles in cancer suppression and promotion. Science.

[22] Burnet M. (1957). Cancer; a biological approach. I. The processes of control. Br. Med. J..

[23] Burnet M. (1957). Cancer: A biological approach. III. Viruses associated with neoplastic conditions. IV. Practical applications. Br. Med. J..

[24] Burnet F.M. (1971). Immunological Surveillance in Neoplasia. Immunol. Rev..

[25] Möller G., Möller E. (1976). The Concept of Immunological Surveillance against Neoplasia. Immunol. Rev..

[26] Thomas L. (1982). On immunosurveillance in human cancer. Yale J. Biol. Med..

[27] Stutman O. (1974). Tumor development after 3-methylcholanthrene in immunologically deficient athymic-nude mice. Science.

[28] Stutman O. (1979). Chemical carcinogenesis in nude mice: Comparison between nude mice from homozygous matings and heterozygous matings and effect of age and carcinogen dose. J. Natl. Cancer Inst..

[29] Stutman O. (1979). Spontaneous tumors in nude mice: Effect of the viable yellow gene. Exp. Cell Biol..

[30] Evans R., Alexander P. (1970). Cooperation of Immune Lymphoid Cells with Macrophages in Tumour Immunity. Nature.

[31] Underwood J.C. (1974). Lymphoreticular infiltration in human tumours: Prognostic and biological implications: A review. Br. J. Cancer.

[32] Gorer P.A., Greenstein J.P., Haddow A. (1956). Some Recent Work on Tumor Immunity. Advances in Cancer Research.

[33] Yona S., Gordon S. (2015). From the Reticuloendothelial to Mononuclear Phagocyte System—The Unaccounted Years. Front. Immunol..

[34] Prehn R.T. (1972). The immune reaction as a stimulator of tumor growth. Science.

[35] Hunig T. (1983). T-cell function and specificity in athymic mice. Immunol. Today.

[36] Gillis S., Union N.A., Baker P.E., Smith K.A. (1979). The in vitro generation and sustained culture of nude mouse cytolytic T-lymphocytes. J. Exp. Med..

[37] Maleckar J.R., Sherman L.A. (1987). The composition of the T cell receptor repertoire in nude mice. J. Immunol..

[38] Dighe A.S., Richards E., Old L.J., Schreiber R.D. (1994). Enhanced in vivo growth and resistance to rejection of tumor cells expressing dominant negative IFN gamma receptors. Immunity.

[39] Smyth M.J., Thia K.Y., Street S.E., MacGregor D., Godfrey D.I., Trapani J.A. (2000). Perforin-mediated cytotoxicity is critical for surveillance of spontaneous lymphoma. J. Exp. Med..

[40] Chen J., Liao S., Xiao Z., Pan Q., Wang X., Shen K., Wang S., Yang L., Guo F., Liu H.F. (2022). The development and improvement of immunodeficient mice and humanized immune system mouse models. Front. Immunol..

[41] Mills C.D., Kincaid K., Alt J.M., Heilman M.J., Hill A.M. (2000). M-1/M-2 macrophages and the Th1/Th2 paradigm. J. Immunol..

[42] Shinkai Y., Rathbun G., Lam K.P., Oltz E.M., Stewart V., Mendelsohn M., Charron J., Datta M., Young F., Stall A.M. (1992). RAG-2-deficient mice lack mature lymphocytes owing to inability to initiate V(D)J rearrangement. Cell.

[43] Kim R., Emi M., Tanabe K. (2007). Cancer immunoediting from immune surveillance to immune escape. Immunology.

[44] Reiman J.M., Kmieciak M., Manjili M.H., Knutson K.L. (2007). Tumor immunoediting and immunosculpting pathways to cancer progression. Semin. Cancer Biol..

[45] Gross E., Sunwoo J.B., Bui J.D. (2013). Cancer immunosurveillance and immunoediting by natural killer cells. Cancer J..

[46] Malmberg K.J., Carlsten M., Björklund A., Sohlberg E., Bryceson Y.T., Ljunggren H.G. (2017). Natural killer cell-mediated immunosurveillance of human cancer. Semin. Immunol..

[47] Dunn G.P., Ikeda H., Bruce A.T., Koebel C., Uppaluri R., Bui J., Chan R., Diamond M., White J.M., Sheehan K.C. (2005). Interferon-gamma and cancer immunoediting. Immunol. Res..

[48] Dunn G.P., Koebel C.M., Schreiber R.D. (2006). Interferons, immunity and cancer immunoediting. Nat. Rev. Immunol..

[49] von Locquenghien M., Rozalén C., Celià-Terrassa T. (2021). Interferons in cancer immunoediting: Sculpting metastasis and immunotherapy response. J. Clin. Investig..

[50] Gocher A.M., Workman C.J., Vignali D.A.A. (2022). Interferon-γ: Teammate or opponent in the tumour microenvironment?. Nat. Rev. Immunol..

[51] Chadwick T.B., So J., Hertzog P.J., Haynes N.M., Parker B.S. (2026). Striking the right balance with type I interferon signalling in cancer. Nat. Rev. Cancer.

[52] Kumar V., Bhat K.H. (2019). Macrophages: The Potent Immunoregulatory Innate Immune Cells. Macrophage Activation-Biology and Disease.

[53] Medzhitov R., Preston-Hurlburt P., Janeway C.A. (1997). A human homologue of the Drosophila Toll protein signals activation of adaptive immunity. Nature.

[54] Janeway C.A. (1989). Approaching the asymptote? Evolution and revolution in immunology. Cold Spring Harb. Symp. Quant. Biol..

[55] Medzhitov R. (2009). Approaching the Asymptote: 20 Years Later. Immunity.

[56] Kumar V., Barrett J.E. (2022). Toll-like Receptors (TLRs) in Health and Disease: An Overview. Toll-like Receptors in Health and Disease.

[57] Kumar V. (2018). Toll-like receptors in immunity and inflammatory diseases: Past, present, and future. Int. Immunopharmacol..

[58] Kumar V. (2021). Going, Toll-like receptors in skin inflammation and inflammatory diseases. EXCLI J..

[59] Kumar V. (2019). Toll-like receptors in the pathogenesis of neuroinflammation. J. Neuroimmunol..

[60] Kumar V. (2024). Toll-like Receptors in Immunity and Inflammation. Thirty Years Since the Discovery of Toll-like Receptors.

[61] Kumar V. (2022). Toll-like Receptors in Adaptive Immunity. Toll-like Receptors in Health and Disease.

[62] Burnet F.M. (1970). The concept of immunological surveillance. Immunological Aspects of Neoplasia.

[63] Ting R.C., Law L.W. (1967). Thymic function and carcinogenesis. Progress in Experimental Tumor Research.

[64] Smyth M.J., Thia K.Y., Street S.E., Cretney E., Trapani J.A., Taniguchi M., Kawano T., Pelikan S.B., Crowe N.Y., Godfrey D.I. (2000). Differential tumor surveillance by natural killer (NK) and NKT cells. J. Exp. Med..

[65] Wiltrout R.H. (2000). Regulation and antimetastatic functions of liver-associated natural killer cells. Immunol. Rev..

[66] Smyth M.J., Godfrey D.I. (2000). NKT cells and tumor immunity--a double-edged sword. Nat. Immunol..

[67] Fultz M.J., Barber S.A., Dieffenbach C.W., Vogel S.N. (1993). Induction of IFN-γ in macrophages by lipopolysaccharide. Int. Immunol..

[68] Kanevskiy L.M., Telford W.G., Sapozhnikov A.M., Kovalenko E.I. (2013). Lipopolysaccharide induces IFN-γ production in human NK cells. Front. Immunol..

[69] Wang P., Han X., Mo B., Huang G., Wang C. (2017). LPS enhances TLR4 expression and IFN-γ production via the TLR4/IRAK/NF-κB signaling pathway in rat pulmonary arterial smooth muscle cells. Mol. Med. Rep..

[70] Herkiloglu D., Gokce S., Cevik O. (2022). Relationship of interferon regulator factor 5 and interferon-γ with missed abortion. Exp. Ther. Med..

[71] Yasuda K., Shukla P., Bonegio R.G., Rifkin I. (2016). IFN Regulatory Factor-5 Signaling Increases IFN-Gamma Production and Suppresses IL-2 Production from CD4+ T Cells, and Controls IgG Production in B Cells. Arthiritis Rheumatol..

[72] Alibashe-Ahmed M., Roger T., Serre-Beinier V., Berishvili E., Reith W., Bosco D., Berney T. (2019). Macrophage migration inhibitory factor regulates TLR4 expression and modulates TCR/CD3-mediated activation in CD4+ T lymphocytes. Sci. Rep..

[73] van den Broek M.E., Kägi D., Ossendorp F., Toes R., Vamvakas S., Lutz W.K., Melief C.J., Zinkernagel R.M., Hengartner H. (1996). Decreased tumor surveillance in perforin-deficient mice. J. Exp. Med..

[74] van den Broek M.F., Hengartner H. (2000). The role of perforin in infections and tumour surveillance. Exp. Physiol..

[75] Girardi M., Oppenheim D.E., Steele C.R., Lewis J.M., Glusac E., Filler R., Hobby P., Sutton B., Tigelaar R.E., Hayday A.C. (2001). Regulation of cutaneous malignancy by gammadelta T cells. Science.

[76] Doherty G.M., Boucher L., Sorenson K., Lowney J. (2001). Interferon regulatory factor expression in human breast cancer. Ann. Surg..

[77] Yim J.H., Ro S.H., Lowney J.K., Wu S.J., Connett J., Doherty G.M. (2003). The role of interferon regulatory factor-1 and interferon regulatory factor-2 in IFN-gamma growth inhibition of human breast carcinoma cell lines. J. Interferon Cytokine Res..

[78] Nguyen H., Hiscott J., Pitha P.M. (1997). The growing family of interferon regulatory factors. Cytokine Growth Factor Rev..

[79] Lukhele S., Rabbo D.A., Guo M., Shen J., Elsaesser H.J., Quevedo R., Carew M., Gadalla R., Snell L.M., Mahesh L. (2022). The transcription factor IRF2 drives interferon-mediated CD8+ T cell exhaustion to restrict anti-tumor immunity. Immunity.

[80] Borden E.S., Castellano D., Kantzos E.A., Hughes A., Leibovit-Reiben Z., Stockard A., Choudhary A., Ma T., Li X., Mangold A.R. (2026). Immunoediting restricts clonal neoantigens in primary, treatment-naive human tumors. Immunity.

[81] Dunn G.P., Old L.J., Schreiber R.D. (2004). The three Es of cancer immunoediting. Annu. Rev. Immunol..

[82] Patras L., Shaashua L., Matei I., Lyden D. (2023). Immune determinants of the pre-metastatic niche. Cancer Cell.

[83] Sceneay J., Parker B.S., Smyth M.J., Möller A. (2013). Hypoxia-driven immunosuppression contributes to the pre-metastatic niche. Oncoimmunology.

[84] Rabas N., Ferreira R.M.M., Di Blasio S., Malanchi I. (2024). Cancer-induced systemic pre-conditioning of distant organs: Building a niche for metastatic cells. Nat. Rev. Cancer.

[85] Hart I.R. (1982). ‘Seed and soil’ revisited: Mechanisms of site-specific metastasis. Cancer Metastasis Rev..

[86] Witz I.P. (2009). The tumor microenvironment: The making of a paradigm. Cancer Microenviron..

[87] Paget S. (1889). The Distribution of Secondary Growths in Cancer of the Breast. Lancet.

[88] Paget S. (1989). The distribution of secondary growths in cancer of the breast. 1889. Cancer Metastasis Rev..

[89] Witz I.P., Levy-Nissenbaum O. (2006). The tumor microenvironment in the post-PAGET era. Cancer Lett..

[90] Weber C.E., Kuo P.C. (2012). The tumor microenvironment. Surg. Oncol..

[91] Anderson N.M., Simon M.C. (2020). The tumor microenvironment. Curr. Biol..

[92] Truffi M., Sorrentino L., Corsi F. (2020). Fibroblasts in the Tumor Microenvironment. Adv. Exp. Med. Biol..

[93] Cheng B., Yu Q., Wang W. (2023). Intimate communications within the tumor microenvironment: Stromal factors function as an orchestra. J. Biomed. Sci..

[94] Lim H., Moon A. (2016). Inflammatory fibroblasts in cancer. Arch. Pharmacal Res..

[95] Spaeth E.L., Dembinski J.L., Sasser A.K., Watson K., Klopp A., Hall B., Andreeff M., Marini F. (2009). Mesenchymal stem cell transition to tumor-associated fibroblasts contributes to fibrovascular network expansion and tumor progression. PLoS ONE.

[96] Silzle T., Randolph G.J., Kreutz M., Kunz-Schughart L.A. (2004). The fibroblast: Sentinel cell and local immune modulator in tumor tissue. Int. J. Cancer.

[97] Mao X., Xu J., Wang W., Liang C., Hua J., Liu J., Zhang B., Meng Q., Yu X., Shi S. (2021). Crosstalk between cancer-associated fibroblasts and immune cells in the tumor microenvironment: New findings and future perspectives. Mol. Cancer.

[98] Ikeda H., Kawase K., Nishi T., Watanabe T., Takenaga K., Inozume T., Ishino T., Aki S., Lin J., Kawashima S. (2025). Immune evasion through mitochondrial transfer in the tumour microenvironment. Nature.

[99] Kumar V., Stewart J.H.t. (2023). Immunometabolic reprogramming, another cancer hallmark. Front. Immunol..

[100] Ward P.S., Thompson C.B. (2012). Metabolic Reprogramming: A Cancer Hallmark Even Warburg Did Not Anticipate. Cancer Cell.

[101] Arner E.N., Rathmell J.C. (2023). Metabolic programming and immune suppression in the tumor microenvironment. Cancer Cell.

[102] Zhang X., Liu D., Yin S., Gao Y., Li X., Wu G. (2025). Metabolism and epigenetics in cancer: Toward personalized treatment. Front. Endocrinol..

[103] Xu X., Peng Q., Jiang X., Tan S., Yang Y., Yang W., Han Y., Chen Y., Oyang L., Lin J. (2023). Metabolic reprogramming and epigenetic modifications in cancer: From the impacts and mechanisms to the treatment potential. Exp. Mol. Med..

[104] Yang J., Xu J., Wang W., Zhang B., Yu X., Shi S. (2023). Epigenetic regulation in the tumor microenvironment: Molecular mechanisms and therapeutic targets. Signal Transduct. Target. Ther..

[105] Baylin S.B., Jones P.A. (2011). A decade of exploring the cancer epigenome—Biological and translational implications. Nat. Rev. Cancer.

[106] Kim T.H., Lim S.H., Lee H., Chae Y.C., Min D.S. (2026). Metabolic crosstalk among cancer-associated fibroblasts, adipocytes and immune cells as an immunosuppressive tumor microenvironment driver. Exp. Mol. Med..

[107] de Visser K.E., Joyce J.A. (2023). The evolving tumor microenvironment: From cancer initiation to metastatic outgrowth. Cancer Cell.

[108] Binnewies M., Roberts E.W., Kersten K., Chan V., Fearon D.F., Merad M., Coussens L.M., Gabrilovich D.I., Ostrand-Rosenberg S., Hedrick C.C. (2018). Understanding the tumor immune microenvironment (TIME) for effective therapy. Nat. Med..

[109] Beatty G.L., Winograd R., Evans R.A., Long K.B., Luque S.L., Lee J.W., Clendenin C., Gladney W.L., Knoblock D.M., Guirnalda P.D. (2015). Exclusion of T Cells From Pancreatic Carcinomas in Mice Is Regulated by Ly6C(low) F4/80(+) Extratumoral Macrophages. Gastroenterology.

[110] Sakai Y., Miyazawa M., Komura T., Yamada T., Nasti A., Yoshida K., Takabatake H., Yamato M., Yamashita T., Yamashita T. (2019). Distinct chemotherapy-associated anti-cancer immunity by myeloid cells inhibition in murine pancreatic cancer models. Cancer Sci..

[111] De Palma M., Venneri M.A., Galli R., Sergi Sergi L., Politi L.S., Sampaolesi M., Naldini L. (2005). Tie2 identifies a hematopoietic lineage of proangiogenic monocytes required for tumor vessel formation and a mesenchymal population of pericyte progenitors. Cancer Cell.

[112] De Palma M., Murdoch C., Venneri M.A., Naldini L., Lewis C.E. (2007). Tie2-expressing monocytes: Regulation of tumor angiogenesis and therapeutic implications. Trends Immunol..

[113] Venneri M.A., De Palma M., Ponzoni M., Pucci F., Scielzo C., Zonari E., Mazzieri R., Doglioni C., Naldini L. (2007). Identification of proangiogenic TIE2-expressing monocytes (TEMs) in human peripheral blood and cancer. Blood.

[114] Hirayama D., Iida T., Nakase H. (2017). The Phagocytic Function of Macrophage-Enforcing Innate Immunity and Tissue Homeostasis. Int. J. Mol. Sci..

[115] Kumar V. (2020). Phagocytosis: Phenotypically Simple Yet a Mechanistically Complex Process. Int. Rev. Immunol..

[116] Hume D.A. (2012). Plenary Perspective: The complexity of constitutive and inducible gene expression in mononuclear phagocytes. J. Leukoc. Biol..

[117] Franklin R.A., Li M.O. (2016). Ontogeny of Tumor-associated Macrophages and Its Implication in Cancer Regulation. Trends Cancer.

[118] Evans R., Alexander P. (1972). Mechanism of Immunologically Specific Killing of Tumour Cells by Macrophages. Nature.

[119] Granger G.A., Weiser R.S. (1964). Homograft target cells: Specific destruction in vitro by contact interaction with immune macrophages. Science.

[120] Granger G.A., Weiser R.S. (1966). Homograft target cells: Contact destruction in vitro by immune macrophages. Science.

[121] Kuntz B.M., Kuntz R.M., Albert E.D. (1978). Phagocytosis of monocytes in cancer patients. Z. Krebsforsch. Klin. Onkol..

[122] Nielsen H., Bennedsen J., Larsen S.O., Dombernowsky P., Viskum K. (1982). A quantitative and qualitative study of blood monocytes in patients with bronchogenic carcinoma. Cancer Immunol. Immunother..

[123] Evans R. (1973). Macrophages and the tumour bearing host. Br. J. Cancer Suppl..

[124] Sokol R.J., Hudson G. (1983). Disordered function of mononuclear phagocytes in malignant disease. J. Clin. Pathol..

[125] Alexander P. (1974). The role of macrophages in tumour immunity. J. Clin. Pathol. Suppl. (R. Coll. Pathol.).

[126] Peng M., Li N., Wang H., Li Y., Liu H., Luo Y., Lang B., Zhang W., Li S., Tian L. (2025). Macrophages: Subtypes, Distribution, Polarization, Immunomodulatory Functions, and Therapeutics. MedComm.

[127] Chang Y., Lei Y., Wang L., Liu L., Yu R. (2026). Decoding the dual roles of monocytes in tumor immunity: From immunosurveillance to immune evasion. Front. Immunol..

[128] Hibbs J.B., Taintor R.R., Vavrin Z. (1987). Macrophage cytotoxicity: Role for L-arginine deiminase and imino nitrogen oxidation to nitrite. Science.

[129] Mao Y., Xia W., Jiang P. (2026). Metabolites as signalling molecules in the tumour immune microenvironment. Nat. Rev. Immunol..

[130] Wynn T.A., Chawla A., Pollard J.W. (2013). Macrophage biology in development, homeostasis and disease. Nature.

[131] Cotechini T., Atallah A., Grossman A. (2021). Tissue-Resident and Recruited Macrophages in Primary Tumor and Metastatic Microenvironments: Potential Targets in Cancer Therapy. Cells.

[132] Christofides A., Strauss L., Yeo A., Cao C., Charest A., Boussiotis V.A. (2022). The complex role of tumor-infiltrating macrophages. Nat. Immunol..

[133] Hibbs J.B., Vavrin Z., Taintor R.R. (1987). L-arginine is required for expression of the activated macrophage effector mechanism causing selective metabolic inhibition in target cells. J. Immunol..

[134] Hibbs J.B., Taintor R.R., Vavrin Z., Rachlin E.M. (1988). Nitric oxide: A cytotoxic activated macrophage effector molecule. Biochem. Biophys. Res. Commun..

[135] Mills C.D., Shearer J., Evans R., Caldwell M.D. (1992). Macrophage arginine metabolism and the inhibition or stimulation of cancer. J. Immunol..

[136] Mills C.D. (1991). Molecular basis of “suppressor” macrophages. Arginine metabolism via the nitric oxide synthetase pathway. J. Immunol..

[137] van der Veen R.C., Dietlin T.A., Dixon Gray J., Gilmore W. (2000). Macrophage-derived nitric oxide inhibits the proliferation of activated T helper cells and is induced during antigenic stimulation of resting T cells. Cell. Immunol..

[138] Sektioglu I.M., Carretero R., Bender N., Bogdan C., Garbi N., Umansky V., Umansky L., Urban K., von Knebel-Döberitz M., Somasundaram V. (2016). Macrophage-derived nitric oxide initiates T-cell diapedesis and tumor rejection. Oncoimmunology.

[139] Fauskanger M., Haabeth O.A.W., Skjeldal F.M., Bogen B., Tveita A.A. (2018). Tumor Killing by CD4+ T Cells Is Mediated via Induction of Inducible Nitric Oxide Synthase-Dependent Macrophage Cytotoxicity. Front. Immunol..

[140] Keshet R., Erez A. (2018). Arginine and the metabolic regulation of nitric oxide synthesis in cancer. Dis. Models Mech..

[141] Kang X., Li H. (2026). Dual Role of Nitric Oxide in Tumor Immunity and Its Therapeutic Application. Front. Biosci. (Landmark Ed.).

[142] Carron E.C., Homra S., Rosenberg J., Coffelt S.B., Kittrell F., Zhang Y., Creighton C.J., Fuqua S.A., Medina D., Machado H.L. (2017). Macrophages promote the progression of premalignant mammary lesions to invasive cancer. Oncotarget.

[143] Casanova-Acebes M., Dalla E., Leader A.M., LeBerichel J., Nikolic J., Morales B.M., Brown M., Chang C., Troncoso L., Chen S.T. (2021). Tissue-resident macrophages provide a pro-tumorigenic niche to early NSCLC cells. Nature.

[144] Russell D.G., Cardona P.J., Kim M.J., Allain S., Altare F. (2009). Foamy macrophages and the progression of the human tuberculosis granuloma. Nat. Immunol..

[145] DuPage M., Dooley A.L., Jacks T. (2009). Conditional mouse lung cancer models using adenoviral or lentiviral delivery of Cre recombinase. Nat. Protoc..

[146] Sutherland K.D., Song J.Y., Kwon M.C., Proost N., Zevenhoven J., Berns A. (2014). Multiple cells-of-origin of mutant K-Ras-induced mouse lung adenocarcinoma. Proc. Natl. Acad. Sci. USA.

[147] De Zuani M., Xue H., Park J.S., Dentro S.C., Seferbekova Z., Tessier J., Curras-Alonso S., Hadjipanayis A., Athanasiadis E.I., Gerstung M. (2024). Single-cell and spatial transcriptomics analysis of non-small cell lung cancer. Nat. Commun..

[148] Haug M., Pettersen H.S., Børset M., Kildahl-Andersen A., Wahl S.G.F., Flatberg A., Tøndell A. (2026). Immune-cold NSCLC tumors harbor tumor-associated macrophages with elevated expression of immunoglobulin genes. Cancer Immunol. Immunother..

[149] Zhu P., Liu Z., Husain H., Wang Q., Zhou Y. (2025). Single-cell and spatial analysis reveals macrophage-T cell crosstalk in non-small cell lung cancer immunosuppression. Transl. Lung Cancer Res..

[150] Pietra G., Romagnani C., Manzini C., Moretta L., Mingari M.C. (2010). The emerging role of HLA-E-restricted CD8+ T lymphocytes in the adaptive immune response to pathogens and tumors. J. Biomed. Biotechnol..

[151] Pietra G., Romagnani C., Moretta L., Mingari M.C. (2009). HLA-E and HLA-E-bound peptides: Recognition by subsets of NK and T cells. Curr. Pharm. Des..

[152] Braud V.M., Allan D.S.J., O’Callaghan C.A., Söderström K., D’Andrea A., Ogg G.S., Lazetic S., Young N.T., Bell J.I., Phillips J.H. (1998). HLA-E binds to natural killer cell receptors CD94/NKG2A, B and C. Nature.

[153] Garrido-Martin E.M., Mellows T.W.P., Clarke J., Ganesan A.P., Wood O., Cazaly A., Seumois G., Chee S.J., Alzetani A., King E.V. (2020). M1(hot) tumor-associated macrophages boost tissue-resident memory T cells infiltration and survival in human lung cancer. J. Immunother. Cancer.

[154] Lavin Y., Kobayashi S., Leader A., Amir E.D., Elefant N., Bigenwald C., Remark R., Sweeney R., Becker C.D., Levine J.H. (2017). Innate Immune Landscape in Early Lung Adenocarcinoma by Paired Single-Cell Analyses. Cell.

[155] Xu X., Li Y., He S., Wen F., Guo N., Wu S. (2025). LC3 overexpression in colorectal cancer—Strong correlation with M1 tumor-associated macrophages: An observational study. Medicine.

[156] Tanida I., Ueno T., Kominami E. (2008). LC3 and Autophagy. Methods Mol. Biol..

[157] Niu X., You Q., Hou K., Tian Y., Wei P., Zhu Y., Gao B., Ashrafizadeh M., Aref A.R., Kalbasi A. (2025). Autophagy in cancer development, immune evasion, and drug resistance. Drug Resist. Updates.

[158] Xia H., Green D.R., Zou W. (2021). Autophagy in tumour immunity and therapy. Nat. Rev. Cancer.

[159] Hume D.A., Freeman T.C. (2014). Transcriptomic analysis of mononuclear phagocyte differentiation and activation. Immunol. Rev..

[160] Murray P.J., Allen J.E., Biswas S.K., Fisher E.A., Gilroy D.W., Goerdt S., Gordon S., Hamilton J.A., Ivashkiv L.B., Lawrence T. (2014). Macrophage Activation and Polarization: Nomenclature and Experimental Guidelines. Immunity.

[161] Gordon S., Plüddemann A. (2017). Tissue macrophages: Heterogeneity and functions. BMC Biol..

[162] Gordon S., Plüddemann A., Martinez Estrada F. (2014). Macrophage heterogeneity in tissues: Phenotypic diversity and functions. Immunol. Rev..

[163] Ammarah U., Pereira-Nunes A., Delfini M., Mazzone M. (2024). From monocyte-derived macrophages to resident macrophages-how metabolism leads their way in cancer. Mol. Oncol..

[164] Han J., Gallerand A., Erlich E.C., Helmink B.A., Mair I., Li X., Eckhouse S.R., Dimou F.M., Shakhsheer B.A., Phelps H.M. (2024). Human serous cavity macrophages and dendritic cells possess counterparts in the mouse with a distinct distribution between species. Nat. Immunol..

[165] Cassetta L., Pollard J.W. (2018). Targeting macrophages: Therapeutic approaches in cancer. Nat. Rev. Drug Discov..

[166] Lahmar Q., Keirsse J., Laoui D., Movahedi K., Van Overmeire E., Van Ginderachter J.A. (2016). Tissue-resident versus monocyte-derived macrophages in the tumor microenvironment. Biochim. Biophys. Acta (BBA)-Rev. Cancer.

[167] Mantovani A., Allavena P., Marchesi F., Garlanda C. (2022). Macrophages as tools and targets in cancer therapy. Nat. Rev. Drug Discov..

[168] Mantovani A., Marchesi F., Malesci A., Laghi L., Allavena P. (2017). Tumour-associated macrophages as treatment targets in oncology. Nat. Rev. Clin. Oncol..

[169] Doig T.N., Hume D.A., Theocharidis T., Goodlad J.R., Gregory C.D., Freeman T.C. (2013). Coexpression analysis of large cancer datasets provides insight into the cellular phenotypes of the tumour microenvironment. BMC Genom..

[170] Chang Y.-C., Chen T.-C., Lee C.-T., Yang C.-Y., Wang H.-W., Wang C.-C., Hsieh S.-L. (2008). Epigenetic control of MHC class II expression in tumor-associated macrophages by decoy receptor 3. Blood.

[171] Yang N., Yuan H., Wang W., Liu X., Liu C., Yao Y., Li Y., Gao Y., Lin X., Wang M. (2026). Pan-cancer macrophage atlas uncovers that MHC-IIhigh TAMs induce Treg activation through physical interactions. Cell Rep..

[172] Jeong M.R., Hwang J.W., Choi M., Seok S.H. (2025). MHC class II+ macrophage differentiation is impaired in metastasized lungs via PGE2 receptor EP2. Cell Rep..

[173] Kuratani A., Okamoto M., Kishida K., Okuzaki D., Sasai M., Sakaguchi S., Arase H., Yamamoto M. (2024). Platelet factor 4–induced TH1-Treg polarization suppresses antitumor immunity. Science.

[174] Moreno Ayala M.A., Campbell T.F., Zhang C., Dahan N., Bockman A., Prakash V., Feng L., Sher T., DuPage M. (2023). CXCR3 expression in regulatory T cells drives interactions with type I dendritic cells in tumors to restrict CD8(+) T cell antitumor immunity. Immunity.

[175] He D., Wang D., Lu P., Yang N., Xue Z., Zhu X., Zhang P., Fan G. (2021). Single-cell RNA sequencing reveals heterogeneous tumor and immune cell populations in early-stage lung adenocarcinomas harboring EGFR mutations. Oncogene.

[176] Bischoff P., Trinks A., Obermayer B., Pett J.P., Wiederspahn J., Uhlitz F., Liang X., Lehmann A., Jurmeister P., Elsner A. (2021). Single-cell RNA sequencing reveals distinct tumor microenvironmental patterns in lung adenocarcinoma. Oncogene.

[177] Pfirrmann M., Bödder J., Wuestenenk R., Tennebroek J., Lyer K.K., Poel D., Tauriello D.V.F., Verdoes M., de Vries I.J.M. (2025). The Phenotypical and Functional Effect of PGE2 on Human Macrophages. Eur. J. Immunol..

[178] MacKenzie K.F., Clark K., Naqvi S., McGuire V.A., Nöehren G., Kristariyanto Y., van den Bosch M., Mudaliar M., McCarthy P.C., Pattison M.J. (2013). PGE(2) induces macrophage IL-10 production and a regulatory-like phenotype via a protein kinase A-SIK-CRTC3 pathway. J. Immunol..

[179] Luan B., Yoon Y.-S., Le Lay J., Kaestner K.H., Hedrick S., Montminy M. (2015). CREB pathway links PGE2 signaling with macrophage polarization. Proc. Natl. Acad. Sci. USA.

[180] Fumet J.-D., Latour C., Nuttin L., Derangère V., Ilie A., Russo P., Hampe L., Daumoine S., Thibaudin M., Truntzer C. (2026). Tumor-Associated Macrophages Produce PGE2 to Promote CD8+ T-cell Exhaustion and Drive Resistance to PD-L1 Blockade in Microsatellite-Stable Colorectal Cancer. Cancer Res..

[181] Masuda T., Sankowski R., Staszewski O., Böttcher C., Amann L., Sagar, Scheiwe C., Nessler S., Kunz P., van Loo G. (2019). Spatial and temporal heterogeneity of mouse and human microglia at single-cell resolution. Nature.

[182] Baumgart M., Moos V., Schuhbauer D., Müller B. (1998). Differential expression of major histocompatibility complex class II genes on murine macrophages associated with T cell cytokine profile and protective/suppressive effects. Proc. Natl. Acad. Sci. USA.

[183] Guillaume J., Leufgen A., Hager F.T., Pabst O., Cerovic V. (2023). MHCII expression on gut macrophages supports T cell homeostasis and is regulated by microbiota and ontogeny. Sci. Rep..

[184] Park M.D., Reyes-Torres I., LeBerichel J., Hamon P., LaMarche N.M., Hegde S., Belabed M., Troncoso L., Grout J.A., Magen A. (2023). TREM2 macrophages drive NK cell paucity and dysfunction in lung cancer. Nat. Immunol..

[185] Molgora M., Liu Y.A., Colonna M., Cella M. (2023). TREM2: A new player in the tumor microenvironment. Semin. Immunol..

[186] Wang Q., Zheng K., Tan D., Liang G. (2022). TREM2 knockdown improves the therapeutic effect of PD-1 blockade in hepatocellular carcinoma. Biochem. Biophys. Res. Commun..

[187] Sun R., Han R., McCornack C., Khan S., Tabor G.T., Chen Y., Hou J., Jiang H., Schoch K.M., Mao D.D. (2023). TREM2 inhibition triggers antitumor cell activity of myeloid cells in glioblastoma. Sci. Adv..

[188] Peshoff M.M., Gupta P., Oberai S., Trivedi R., Katayama H., Chakrapani P., Dang M., Migliozzi S., Gumin J., Kadri D.B. (2024). Triggering receptor expressed on myeloid cells 2 (TREM2) regulates phagocytosis in glioblastoma. Neuro Oncol..

[189] Ross J.L., Puigdelloses-Vallcorba M., Piñero G., Soni N., Thomason W., DeSisto J., Angione A., Tsankova N.M., Castro M.G., Schniederjan M. (2024). Microglia and monocyte-derived macrophages drive progression of pediatric high-grade gliomas and are transcriptionally shaped by histone mutations. Immunity.

[190] Zhang C.R., Xiong L., Gu M., Yeh C.Y., Jerby L., Kundaje A., Greenleaf W.J., Bassik M.C. (2025). Cancer cell phagocytosis induces an anti-inflammatory gene regulatory program in macrophages. bioRxiv.

[191] Gonzalez-Kozlova E., Sweeney R., Figueiredo I., Tuballes K., Ozbey S., Hamon P., Park M.D., Ioannou G., Nose Y., Guo R. (2026). Humoral IgG1 responses to tumor antigens underpin clinical outcomes in immune checkpoint blockade. Nat. Med..

[192] Astuti Y., Raymant M., Quaranta V., Clarke K., Abudula M., Smith O., Bellomo G., Chandran-Gorner V., Nourse C., Halloran C. (2024). Efferocytosis reprograms the tumor microenvironment to promote pancreatic cancer liver metastasis. Nat. Cancer.

[193] Nielsen S.R., Quaranta V., Linford A., Emeagi P., Rainer C., Santos A., Ireland L., Sakai T., Sakai K., Kim Y.-S. (2016). Macrophage-secreted granulin supports pancreatic cancer metastasis by inducing liver fibrosis. Nat. Cell Biol..

[194] Raymant M., Astuti Y., Alvaro-Espinosa L., Green D., Quaranta V., Bellomo G., Glenn M., Chandran-Gorner V., Palmer D.H., Halloran C. (2024). Macrophage-fibroblast JAK/STAT dependent crosstalk promotes liver metastatic outgrowth in pancreatic cancer. Nat. Commun..

[195] Liao C.-Y., Li G., Kang F.-P., Lin C.-F., Xie C.-K., Wu Y.-D., Hu J.-F., Lin H.-Y., Zhu S.-C., Huang X.-X. (2024). Necroptosis enhances ‘don’t eat me’ signal and induces macrophage extracellular traps to promote pancreatic cancer liver metastasis. Nat. Commun..

[196] Franklin R.A., Liao W., Sarkar A., Kim M.V., Bivona M.R., Liu K., Pamer E.G., Li M.O. (2014). The cellular and molecular origin of tumor-associated macrophages. Science.

[197] Choi J., Gyamfi J., Jang H., Koo J.S. (2018). The role of tumor-associated macrophage in breast cancer biology. Histol. Histopathol..

[198] Yin Z.-Q., Wen T., Cao X.-L., Lv Z.-X., Su Y., Liang L., Zhang P.-R., Yang Z.-Y., Han H., Yan X.-C. (2025). The transcription factor RBPJ is required for inflammatory macrophage activation in thoracic aortic dissection by mediating mechanotransduction-induced glycolysis. Cell. Mol. Life Sci..

[199] Wang Y.C., He F., Feng F., Liu X.W., Dong G.Y., Qin H.Y., Hu X.B., Zheng M.H., Liang L., Feng L. (2010). Notch signaling determines the M1 versus M2 polarization of macrophages in antitumor immune responses. Cancer Res..

[200] Foldi J., Shang Y., Zhao B., Ivashkiv L.B., Hu X. (2016). RBP-J is required for M2 macrophage polarization in response to chitin and mediates expression of a subset of M2 genes. Protein Cell.

[201] Lang R., Pauleau A.-L., Parganas E., Takahashi Y., Mages J., Ihle J.N., Rutschman R., Murray P.J. (2003). SOCS3 regulates the plasticity of gp130 signaling. Nat. Immunol..

[202] Qin H., Holdbrooks A.T., Liu Y., Reynolds S.L., Yanagisawa L.L., Benveniste E.N. (2012). SOCS3 deficiency promotes M1 macrophage polarization and inflammation. J. Immunol..

[203] Coffelt S.B., Tal A.O., Scholz A., De Palma M., Patel S., Urbich C., Biswas S.K., Murdoch C., Plate K.H., Reiss Y. (2010). Angiopoietin-2 regulates gene expression in TIE2-expressing monocytes and augments their inherent proangiogenic functions. Cancer Res..

[204] Chen S., Huang C., Li K., Cheng M., Zhang C., Xiong J., Tian G., Zhou R., Ling R., Wang X. (2025). Tumor-initiating cells escape tumor immunity via CCL8 from tumor-associated macrophages in mice. J. Clin. Investig..

[205] Chavez B., Kiaris H. (2025). Insights on the role of the chemokine CCL8 in pathology. Cell. Signal..

[206] Zhang X., Chen L., Dang W.Q., Cao M.F., Xiao J.F., Lv S.Q., Jiang W.J., Yao X.H., Lu H.M., Miao J.Y. (2020). CCL8 secreted by tumor-associated macrophages promotes invasion and stemness of glioblastoma cells via ERK1/2 signaling. Lab. Investig..

[207] Waibl Polania J., Hoyt-Miggelbrink A., Tomaszewski W.H., Wachsmuth L.P., Lorrey S.J., Wilkinson D.S., Lerner E., Woroniecka K., Finlay J.B., Ayasoufi K. (2025). Antigen presentation by tumor-associated macrophages drives T cells from a progenitor exhaustion state to terminal exhaustion. Immunity.

[208] Fusilier Z., Clément A., Simon F., Calvente I., Jean-Marie R., Mathieu M., Calmettes V., Piastra-Facon F., Quintana-Perez Y., Clement J.G. (2026). Macrophages restrict tumor immune infiltration by controlling collagen topography. Sci. Immunol..

[209] Belgiovine C., Digifico E., Anfray C., Ummarino A., Torres Andón F. (2020). Targeting Tumor-Associated Macrophages in Anti-Cancer Therapies: Convincing the Traitors to Do the Right Thing. J. Clin. Med..

[210] Kzhyshkowska J., Shen J., Larionova I. (2024). Targeting of TAMs: Can we be more clever than cancer cells?. Cell. Mol. Immunol..

[211] Rannikko J.H., Hollmén M. (2024). Clinical landscape of macrophage-reprogramming cancer immunotherapies. Br. J. Cancer.

[212] Sun X., Park M.D., Merad M., Brown B.D. (2026). Macrophages: Targets for next-generation cancer immunotherapy. Cancer Cell.

[213] Cassetta L., Pollard J.W. (2023). A timeline of tumour-associated macrophage biology. Nat. Rev. Cancer.

[214] Pittet M.J., Michielin O., Migliorini D. (2022). Clinical relevance of tumour-associated macrophages. Nat. Rev. Clin. Oncol..

[215] Ruffell B., Coussens L.M. (2015). Macrophages and therapeutic resistance in cancer. Cancer Cell.

[216] Noy R., Pollard J.W. (2014). Tumor-associated macrophages: From mechanisms to therapy. Immunity.

[217] Kloosterman D.J., Akkari L. (2023). Macrophages at the interface of the co-evolving cancer ecosystem. Cell.

[218] Nasir I., McGuinness C., Poh A.R., Ernst M., Darcy P.K., Britt K.L. (2023). Tumor macrophage functional heterogeneity can inform the development of novel cancer therapies. Trends Immunol..

[219] Klichinsky M., Ruella M., Shestova O., Lu X.M., Best A., Zeeman M., Schmierer M., Gabrusiewicz K., Anderson N.R., Petty N.E. (2020). Human chimeric antigen receptor macrophages for cancer immunotherapy. Nat. Biotechnol..

[220] Kingwell K. (2017). CAR T therapies drive into new terrain. Nat. Rev. Drug Discov..

[221] Maude S.L., Laetsch T.W., Buechner J., Rives S., Boyer M., Bittencourt H., Bader P., Verneris M.R., Stefanski H.E., Myers G.D. (2018). Tisagenlecleucel in Children and Young Adults with B-Cell Lymphoblastic Leukemia. N. Engl. J. Med..

[222] Schuster S.J., Svoboda J., Chong E.A., Nasta S.D., Mato A.R., Anak Ö., Brogdon J.L., Pruteanu-Malinici I., Bhoj V., Landsburg D. (2017). Chimeric Antigen Receptor T Cells in Refractory B-Cell Lymphomas. N. Engl. J. Med..

[223] Dana H., Chalbatani G.M., Jalali S.A., Mirzaei H.R., Grupp S.A., Suarez E.R., Rapôso C., Webster T.J. (2021). CAR-T cells: Early successes in blood cancer and challenges in solid tumors. Acta Pharm. Sin. B.

[224] Ziane-Chaouche L., Raffo-Romero A., Hajjaji N., Kobeissy F., Pinheiro D., Aboulouard S., Cozzani A., Mitra S., Fournier I., Cizkova D. (2024). Inhibition of furin in CAR macrophages directs them toward a proinflammatory phenotype and enhances their antitumor activities. Cell Death Dis..

[225] Alrehaili M., Couto P.S., Khalife R., Rafiq Q.A. (2025). CAR-macrophages in solid tumors: Promise, progress, and prospects. npj Precis. Oncol..

[226] Lei A., Yu H., Lu S., Lu H., Ding X., Tan T., Zhang H., Zhu M., Tian L., Wang X. (2024). A second-generation M1-polarized CAR macrophage with antitumor efficacy. Nat. Immunol..

[227] Hong J., Lee S., Kim Y., Kang C.K., Park W.B., Shin H.M., Kim H.-R., Lee C.-H. (2026). Optimization of THP-1-CAR monocytes utilizing CD32a signaling phagocytosis for antigen-specific T cell activation. Sci. Rep..

[228] Reiss K.A., Angelos M.G., Dees E.C., Yuan Y., Ueno N.T., Pohlmann P.R., Johnson M.L., Chao J., Shestova O., Serody J.S. (2025). CAR-macrophage therapy for HER2-overexpressing advanced solid tumors: A phase 1 trial. Nat. Med..

[229] Shah Z., Tian L., Li Z., Jin L., Zhang J., Li Z., Barr T., Tang H., Feng M., Caligiuri M.A. (2024). Human anti-PSCA CAR macrophages possess potent antitumor activity against pancreatic cancer. Cell Stem Cell.

[230] Kang M., Lee S.H., Kwon M., Byun J., Kim D., Kim C., Koo S., Kwon S.P., Moon S., Jung M. (2021). Nanocomplex-Mediated In Vivo Programming to Chimeric Antigen Receptor-M1 Macrophages for Cancer Therapy. Adv. Mater..

[231] Paasch D., Lachmann N. (2024). CAR macrophages tuning the immune symphony of anti-cancer therapies. Cell Stem Cell.

[232] Pan K., Farrukh H., Chittepu V.C.S.R., Xu H., Pan C.-x., Zhu Z. (2022). CAR race to cancer immunotherapy: From CAR T, CAR NK to CAR macrophage therapy. J. Exp. Clin. Cancer Res..

[233] Lu J., Ma Y., Li Q., Xu Y., Xue Y., Xu S. (2024). CAR Macrophages: A promising novel immunotherapy for solid tumors and beyond. Biomark. Res..

[234] Chen S., Wang Y., Dang J., Song N., Chen X., Wang J., Huang G.N., Brown C.E., Yu J., Weissman I.L. (2025). CAR macrophages with built-In CD47 blocker combat tumor antigen heterogeneity and activate T cells via cross-presentation. Nat. Commun..

[235] Zhang H., Huo Y., Zheng W., Li P., Li H., Zhang L., Sa L., He Y., Zhao Z., Shi C. (2024). Silencing of SIRPα enhances the antitumor efficacy of CAR-M in solid tumors. Cell. Mol. Immunol..

[236] Huang C., Wang X., Wang Y., Feng Y., Wang X., Chen S., Yan P., Liao J., Zhang Q., Mao C. (2024). Sirpα on tumor-associated myeloid cells restrains antitumor immunity in colorectal cancer independent of its interaction with CD47. Nat. Cancer.

[237] Yamada-Hunter S.A., Theruvath J., McIntosh B.J., Freitas K.A., Lin F., Radosevich M.T., Leruste A., Dhingra S., Martinez-Velez N., Xu P. (2024). Engineered CD47 protects T cells for enhanced antitumour immunity. Nature.

[238] Ha J., Wang Y., Ma Y., Wu A., Dong S., Jeong S.D., Edwards J.L., Chang M., Chang Y.-T., Wang X. (2026). Dual phagocytosis-checkpoint blockade revitalizes immune surveillance in mouse models of glioblastoma. Nat. Commun..

[239] Mishell R.I., Dutton R.W. (1966). Immunization of normal mouse spleen cell suspensions in vitro. Science.

[240] Mosier D.E. (1967). A requirement for two cell types for antibody formation in vitro. Science.

[241] Steinman R.M., Cohn Z.A. (1973). Identification of a novel cell type in peripheral lymphoid organs of mice. I. Morphology, quantitation, tissue distribution. J. Exp. Med..

[242] Steinman R.M., Cohn Z.A. (1974). Identification of a novel cell type in peripheral lymphoid organs of mice. II. Functional properties in vitro. J. Exp. Med..

[243] Steinman R.M., Kaplan G., Witmer M.D., Cohn Z.A. (1979). Identification of a novel cell type in peripheral lymphoid organs of mice. V. Purification of spleen dendritic cells, new surface markers, and maintenance in vitro. J. Exp. Med..

[244] Steinman R.M., Witmer M.D. (1978). Lymphoid dendritic cells are potent stimulators of the primary mixed leukocyte reaction in mice. Proc. Natl. Acad. Sci. USA.

[245] Nussenzweig M.C., Steinman R.M. (1980). Contribution of dendritic cells to stimulation of the murine syngeneic mixed leukocyte reaction. J. Exp. Med..

[246] Katsnelson A. (2006). Kicking off adaptive immunity: The discovery of dendritic cells. J. Exp. Med..

[247] Nussenzweig M.C., Steinman R.M., Gutchinov B., Cohn Z.A. (1980). Dendritic cells are accessory cells for the development of anti-trinitrophenyl cytotoxic T lymphocytes. J. Exp. Med..

[248] Inaba K., Steinman R.M., Van Voorhis W.C., Muramatsu S. (1983). Dendritic cells are critical accessory cells for thymus-dependent antibody responses in mouse and in man. Proc. Natl. Acad. Sci. USA.

[249] Schuler G., Steinman R.M. (1985). Murine epidermal Langerhans cells mature into potent immunostimulatory dendritic cells in vitro. J. Exp. Med..

[250] Romani N., Koide S., Crowley M., Witmer-Pack M., Livingstone A.M., Fathman C.G., Inaba K., Steinman R.M. (1989). Presentation of exogenous protein antigens by dendritic cells to T cell clones. Intact protein is presented best by immature, epidermal Langerhans cells. J. Exp. Med..

[251] Van Voorhis W.C., Valinsky J., Hoffman E., Luban J., Hair L.S., Steinman R.M. (1983). Relative efficacy of human monocytes and dendritic cells as accessory cells for T cell replication. J. Exp. Med..

[252] Klinkert W.E., LaBadie J.H., Bowers W.E. (1982). Accessory and stimulating properties of dendritic cells and macrophages isolated from various rat tissues. J. Exp. Med..

[253] Eisenbarth S.C. (2019). Dendritic cell subsets in T cell programming: Location dictates function. Nat. Rev. Immunol..

[254] Morse M.A., Lyerly H.K. (1998). Immunotherapy of cancer using dendritic cells. Cytokines Cell Mol. Ther..

[255] Tjoa B.A., Murphy G.P. (2000). Development of dendritic-cell based prostate cancer vaccine. Immunol. Lett..

[256] Ribas A., Butterfield L.H., Glaspy J.A., Economou J.S. (2002). Cancer immunotherapy using gene-modified dendritic cells. Curr. Gene Ther..

[257] Palucka A.K., Dhodapkar M.V., Paczesny S., Burkeholder S., Wittkowski K.M., Steinman R.M., Fay J., Banchereau J. (2003). Single Injection of CD34+ Progenitor-Derived Dendritic Cell Vaccine Can Lead to Induction of T-Cell Immunity in Patients with Stage IV Melanoma. J. Immunother..

[258] Nestle F.O., Alijagic S., Gilliet M., Sun Y., Grabbe S., Dummer R., Burg G., Schadendorf D. (1998). Vaccination of melanoma patients with peptide- or tumorlysate-pulsed dendritic cells. Nat. Med..

[259] Thurner B., Haendle I., Röder C., Dieckmann D., Keikavoussi P., Jonuleit H., Bender A., Maczek C., Schreiner D., von den Driesch P. (1999). Vaccination with Mage-3a1 Peptide–Pulsed Mature, Monocyte-Derived Dendritic Cells Expands Specific Cytotoxic T Cells and Induces Regression of Some Metastases in Advanced Stage IV Melanoma. J. Exp. Med..

[260] Nathan C. (2011). Ralph Steinman 1943–2011. Nat. Immunol..

[261] Wang R., Chen Y., Fang Y.-f. (2026). Dendritic cell diversity and dysfunction in cancer: Implications for immunotherapy and therapeutic targeting. Acta Pharmacol. Sin..

[262] Moon C.Y., Belabed M., Park M.D., Mattiuz R., Puleston D., Merad M. (2025). Dendritic cell maturation in cancer. Nat. Rev. Cancer.

[263] De Martino M., Rathmell J.C., Galluzzi L., Vanpouille-Box C. (2024). Cancer cell metabolism and antitumour immunity. Nat. Rev. Immunol..

[264] Espinosa-Carrasco G., Chiu E., Scrivo A., Zumbo P., Dave A., Betel D., Kang S.W., Jang H.J., Hellmann M.D., Burt B.M. (2024). Intratumoral immune triads are required for immunotherapy-mediated elimination of solid tumors. Cancer Cell.

[265] Garris C.S., Arlauckas S.P., Kohler R.H., Trefny M.P., Garren S., Piot C., Engblom C., Pfirschke C., Siwicki M., Gungabeesoon J. (2018). Successful Anti-PD-1 Cancer Immunotherapy Requires T Cell-Dendritic Cell Crosstalk Involving the Cytokines IFN-γ and IL-12. Immunity.

[266] Papadas A., Deb G., Cicala A., Officer A., Hope C., Pagenkopf A., Flietner E., Morrow Z.T., Emmerich P., Wiesner J. (2022). Stromal remodeling regulates dendritic cell abundance and activity in the tumor microenvironment. Cell Rep..

[267] Lim K.H.J., Schulz O., Lobon I., Castro-Dopico T., Zapata L., Giampazolias E., Frederico B., Castellanos C.A., Buck M.D., Stainier W. (2026). Cross-presentation of dead cell-associated antigens shapes the neoantigenic landscape of tumor immunity. Nat. Immunol..

[268] Guo C., You Z., Shi H., Sun Y., Du X., Palacios G., Guy C., Yuan S., Chapman N.M., Lim S.A. (2023). SLC38A2 and glutamine signalling in cDC1s dictate anti-tumour immunity. Nature.

[269] Samie M., Cresswell P. (2015). The transcription factor TFEB acts as a molecular switch that regulates exogenous antigen-presentation pathways. Nat. Immunol..

[270] Lei X., Khatri I., de Wit T., de Rink I., Nieuwland M., Kerkhoven R., van Eenennaam H., Sun C., Garg A.D., Borst J. (2023). CD4+ helper T cells endow cDC1 with cancer-impeding functions in the human tumor micro-environment. Nat. Commun..

[271] Lei X., de Groot D.C., Welters M.J.P., de Wit T., Schrama E., van Eenennaam H., Santegoets S.J., Oosenbrug T., van der Veen A., Vos J.L. (2024). CD4+ T cells produce IFN-I to license cDC1s for induction of cytotoxic T-cell activity in human tumors. Cell. Mol. Immunol..

[272] Ferris S.T., Durai V., Wu R., Theisen D.J., Ward J.P., Bern M.D., Davidson J.T.t., Bagadia P., Liu T., Briseño C.G. (2020). cDC1 prime and are licensed by CD4(+) T cells to induce anti-tumour immunity. Nature.

[273] Wu R., Ohara R.A., Jo S., Liu T.-T., Ferris S.T., Ou F., Kim S., Theisen D.J., Anderson D.A., Wong B.W. (2022). Mechanisms of CD40-dependent cDC1 licensing beyond costimulation. Nat. Immunol..

[274] Kruse B., Buzzai A.C., Shridhar N., Braun A.D., Gellert S., Knauth K., Pozniak J., Peters J., Dittmann P., Mengoni M. (2023). CD4+ T cell-induced inflammatory cell death controls immune-evasive tumours. Nature.

[275] Peterson E.E., Barry K.C. (2020). The Natural Killer-Dendritic Cell Immune Axis in Anti-Cancer Immunity and Immunotherapy. Front. Immunol..

[276] Bödder J., Zahan T., van Slooten R., Schreibelt G., de Vries I.J.M., Flórez-Grau G. (2020). Harnessing the cDC1-NK Cross-Talk in the Tumor Microenvironment to Battle Cancer. Front. Immunol..

[277] Pittet M.J., Di Pilato M., Garris C., Mempel T.R. (2023). Dendritic cells as shepherds of T cell immunity in cancer. Immunity.

[278] Barry K.C., Hsu J., Broz M.L., Cueto F.J., Binnewies M., Combes A.J., Nelson A.E., Loo K., Kumar R., Rosenblum M.D. (2018). A natural killer-dendritic cell axis defines checkpoint therapy-responsive tumor microenvironments. Nat. Med..

[279] Momenilandi M., Lévy R., Sobrino S., Li J., Lagresle-Peyrou C., Esmaeilzadeh H., Fayand A., Le Floc’h C., Guérin A., Della Mina E. (2024). FLT3L governs the development of partially overlapping hematopoietic lineages in humans and mice. Cell.

[280] Böttcher J.P., Bonavita E., Chakravarty P., Blees H., Cabeza-Cabrerizo M., Sammicheli S., Rogers N.C., Sahai E., Zelenay S., Reis e Sousa C. (2018). NK Cells Stimulate Recruitment of cDC1 into the Tumor Microenvironment Promoting Cancer Immune Control. Cell.

[281] Bayerl F., Meiser P., Donakonda S., Hirschberger A., Lacher S.B., Pedde A.M., Hermann C.D., Elewaut A., Knolle M., Ramsauer L. (2023). Tumor-derived prostaglandin E2 programs cDC1 dysfunction to impair intratumoral orchestration of anti-cancer T cell responses. Immunity.

[282] Hato L., Vizcay A., Eguren I., Pérez-Gracia J.L., Rodríguez J., Gállego Pérez-Larraya J., Sarobe P., Inogés S., Díaz de Cerio A.L., Santisteban M. (2024). Dendritic Cells in Cancer Immunology and Immunotherapy. Cancers.

[283] Santisteban M., Solans B.P., Hato L., Urrizola A., Mejías L.D., Salgado E., Sánchez-Bayona R., Toledo E., Rodríguez-Spiteri N., Olartecoechea B. (2021). Final results regarding the addition of dendritic cell vaccines to neoadjuvant chemotherapy in early HER2-negative breast cancer patients: Clinical and translational analysis. Ther. Adv. Med. Oncol..

[284] Mejías Sosa L., López-Janeiro Á., Córdoba Iturriagagoitia A., Sala P., Solans B.P., Hato L., Inogés S., López-Díaz de Cerio A., Guillén-Grima F., Espinós J. (2023). Modification of Breast Cancer Milieu with Chemotherapy plus Dendritic Cell Vaccine: An Approach to Select Best Therapeutic Strategies. Biomedicines.

[285] Kantoff Philip W., Higano Celestia S., Shore Neal D., Berger E.R., Small Eric J., Penson David F., Redfern Charles H., Ferrari Anna C., Dreicer R., Sims Robert B. (2010). Sipuleucel-T Immunotherapy for Castration-Resistant Prostate Cancer. N. Engl. J. Med..

[286] Anassi E., Ndefo U.A. (2011). Sipuleucel-T (provenge) injection: The first immunotherapy agent (vaccine) for hormone-refractory prostate cancer. Pharm. Ther..

[287] Wei X.X., Fong L., Small E.J. (2015). Prostate Cancer Immunotherapy with Sipuleucel-T: Current Standards and Future Directions. Expert Rev. Vaccines.

[288] Murphy T.L., Murphy K.M. (2022). Dendritic cells in cancer immunology. Cell. Mol. Immunol..

[289] Heras-Murillo I., Adán-Barrientos I., Galán M., Wculek S.K., Sancho D. (2024). Dendritic cells as orchestrators of anticancer immunity and immunotherapy. Nat. Rev. Clin. Oncol..

[290] Paczesny S., Ueno H., Fay J., Banchereau J., Palucka A.K. (2003). Dendritic cells as vectors for immunotherapy of cancer. Semin. Cancer Biol..

[291] Dallal R.M., Lotze M.T. (2000). The dendritic cell and human cancer vaccines. Curr. Opin. Immunol..

[292] Gardner A., Ruffell B. (2016). Dendritic Cells and Cancer Immunity. Trends Immunol..

[293] Kvedaraite E., Ginhoux F. (2022). Human dendritic cells in cancer. Sci. Immunol..

[294] Matsuo K., Yoshie O., Kitahata K., Kamei M., Hara Y., Nakayama T. (2021). Recent Progress in Dendritic Cell-Based Cancer Immunotherapy. Cancers.

[295] Wculek S.K., Cueto F.J., Mujal A.M., Melero I., Krummel M.F., Sancho D. (2020). Dendritic cells in cancer immunology and immunotherapy. Nat. Rev. Immunol..

[296] Abascal J., Oh M.S., Liclican E.L., Dubinett S.M., Salehi-Rad R., Liu B. (2023). Dendritic Cell Vaccination in Non-Small Cell Lung Cancer: Remodeling the Tumor Immune Microenvironment. Cells.

[297] Xie Z., Fang Y., Zhang X., Fang Y., Li R., Guo Y., Yang Y., Yang S., Song L. (2025). The emerging role of dendritic cells in the tumor microenvironment: From antigen presentation to targeted immunotherapy. Cell Death Dis..

[298] Sheykhhasan M., Ahmadieh-Yazdi A., Heidari R., Chamanara M., Akbari M., Poondla N., Yang P., Malih S., Manoochehri H., Tanzadehpanah H. (2025). Revolutionizing cancer treatment: The power of dendritic cell-based vaccines in immunotherapy. Biomed. Pharmacother..

[299] Kureshi C.T.S., Walsh M.J., Kureshi R., Cardot-Ruffino V., Agardy D.A., Ali L.R., Dougan J.M., Qiang L., Shen J., Zuo C. (2026). Dendritic cell redundancy enables priming of anti-tumor CD4(+) T cells in pancreatic cancer. Cancer Cell.

[300] Weledji E.P., Enoworock G., Mokake M., Sinju M. (2016). How Grim is Pancreatic Cancer?. Oncol. Rev..

[301] Ascic E., Åkerström F., Sreekumar Nair M., Rosa A., Kurochkin I., Zimmermannova O., Catena X., Rotankova N., Veser C., Rudnik M. (2024). In vivo dendritic cell reprogramming for cancer immunotherapy. Science.

[302] Morante-Palacios O., Fondelli F., Ballestar E., Martínez-Cáceres E.M. (2021). Tolerogenic Dendritic Cells in Autoimmunity and Inflammatory Diseases. Trends Immunol..

[303] Domogalla M.P., Rostan P.V., Raker V.K., Steinbrink K. (2017). Tolerance through Education: How Tolerogenic Dendritic Cells Shape Immunity. Front. Immunol..

[304] Plebanek M.P., Xue Y., Nguyen Y.-V., DeVito N.C., Wang X., Holtzhausen A., Beasley G.M., Theivanthiran B., Hanks B.A. (2024). A lactate-SREBP2 signaling axis drives tolerogenic dendritic cell maturation and promotes cancer progression. Sci. Immunol..

[305] Wang X., Plebanek M.P., Nguyen Y.-V., Bazaz M.R., Sturdivant M., Theivanthiran B., Hanks B.A. (2026). Tumor-derived Extracellular Vesicles Induce ER Stress to Drive Tolerogenic Dendritic Cell Development in the Tumor Microenvironment. bioRxiv.

[306] Zhao F., Xiao C., Evans K.S., Theivanthiran T., DeVito N., Holtzhausen A., Liu J., Liu X., Boczkowski D., Nair S. (2018). Paracrine Wnt5a-β-Catenin Signaling Triggers a Metabolic Program that Drives Dendritic Cell Tolerization. Immunity.

[307] Stone T.W., Williams R.O. (2023). Modulation of T cells by tryptophan metabolites in the kynurenine pathway. Trends Pharmacol. Sci..

[308] Norian L.A., Rodriguez P.C., O’Mara L.A., Zabaleta J., Ochoa A.C., Cella M., Allen P.M. (2009). Tumor-infiltrating regulatory dendritic cells inhibit CD8+ T cell function via L-arginine metabolism. Cancer Res..

[309] Ishigami S., Natsugoe S., Miyazono F., Tokuda K., Nakajo A., Matsumoto M., Okumura H., Nakashima S., Hokita S., Maruyama I. (2004). CD3 zeta expression of regional lymph node and peripheral blood lymphocytes in gastric cancer. Anticancer Res..

[310] Zea A.H., Rodriguez P.C., Culotta K.S., Hernandez C.P., DeSalvo J., Ochoa J.B., Park H.J., Zabaleta J., Ochoa A.C. (2004). L-Arginine modulates CD3zeta expression and T cell function in activated human T lymphocytes. Cell. Immunol..

[311] Kenkel J.A., Tseng W.W., Davidson M.G., Tolentino L.L., Choi O., Bhattacharya N., Seeley E.S., Winer D.A., Reticker-Flynn N.E., Engleman E.G. (2017). An Immunosuppressive Dendritic Cell Subset Accumulates at Secondary Sites and Promotes Metastasis in Pancreatic Cancer. Cancer Res..

[312] Ghiringhelli F., Puig P.E., Roux S., Parcellier A., Schmitt E., Solary E., Kroemer G., Martin F., Chauffert B., Zitvogel L. (2005). Tumor cells convert immature myeloid dendritic cells into TGF-beta-secreting cells inducing CD4+CD25+ regulatory T cell proliferation. J. Exp. Med..

[313] Gabrilovich D. (2004). Mechanisms and functional significance of tumour-induced dendritic-cell defects. Nat. Rev. Immunol..

[314] Chowdhury F.Z., Ramos H.J., Davis L.S., Forman J., Farrar J.D. (2011). IL-12 selectively programs effector pathways that are stably expressed in human CD8+ effector memory T cells in vivo. Blood.

[315] Ramos H.J., Davis A.M., Cole A.G., Schatzle J.D., Forman J., Farrar J.D. (2009). Reciprocal responsiveness to interleukin-12 and interferon-α specifies human CD8+ effector versus central memory T-cell fates. Blood.

[316] Yadav M.K., Singh S.P., Egwuagu C.E. (2025). IL-6/IL-12 superfamily of cytokines and regulatory lymphocytes play critical roles in the etiology and suppression of CNS autoimmune diseases. Front. Immunol..

[317] Korn T., Hiltensperger M. (2021). Role of IL-6 in the commitment of T cell subsets. Cytokine.

[318] Shemesh A., Pickering H., Roybal K.T., Lanier L.L. (2022). Differential IL-12 signaling induces human natural killer cell activating receptor-mediated ligand-specific expansion. J. Exp. Med..

[319] Kirchhammer N., Trefny M.P., Natoli M., Brücher D., Smith S.N., Werner F., Koch V., Schreiner D., Bartoszek E., Buchi M. (2021). Successful IL-12 cancer immunotherapy requires NK cell-derived CCL5 for anti-tumor DC-T cell crosstalk. bioRxiv.

[320] Nish S.A., Schenten D., Wunderlich F.T., Pope S.D., Gao Y., Hoshi N., Yu S., Yan X., Lee H.K., Pasman L. (2014). T cell-intrinsic role of IL-6 signaling in primary and memory responses. eLife.

[321] Böttcher J.P., Schanz O., Garbers C., Zaremba A., Hegenbarth S., Kurts C., Beyer M., Schultze J.L., Kastenmüller W., Rose-John S. (2014). IL-6 *trans*-Signaling-Dependent Rapid Development of Cytotoxic CD8+ T Cell Function. Cell Rep..

[322] Sriburi R., Jackowski S., Mori K., Brewer J.W. (2004). XBP1: A link between the unfolded protein response, lipid biosynthesis, and biogenesis of the endoplasmic reticulum. J. Cell Biol..

[323] Sun X., Gao Y., Meng K., Wang Y., Wang B. (2026). Identification of tolerogenic dendritic cells-related prognostic biomarkers in Wilms tumor via machine learning integration. Discov. Oncol..

[324] DeVito N.C., Plebanek M.P., Theivanthiran B., Hanks B.A. (2019). Role of Tumor-Mediated Dendritic Cell Tolerization in Immune Evasion. Front. Immunol..

[325] Hurwitz A.A., Watkins S.K. (2012). Immune suppression in the tumor microenvironment: A role for dendritic cell-mediated tolerization of T cells. Cancer Immunol. Immunother..

[326] Wang Y., Koyama-Nasu R., Endo Y., Hasegawa I., Onodera A., Chen M., Hirahara K., Motohashi S., Nakayama T., Kimura M.Y. (2026). Distinct thymic pDC populations promote tumor immune tolerance through complementary mechanisms. Sci. Adv..

[327] Wu B., Zhang B., Li B., Wu H., Jiang M. (2024). Cold and hot tumors: From molecular mechanisms to targeted therapy. Signal Transduct. Target. Ther..

[328] Zhang J., Huang D., Saw P.E., Song E. (2022). Turning cold tumors hot: From molecular mechanisms to clinical applications. Trends Immunol..

[329] Sang M., Ge J., Ge J., Tang G., Wang Q., Wu J., Mao L., Ding X., Zhou X. (2025). Immune regulatory genes impact the hot/cold tumor microenvironment, affecting cancer treatment and patient outcomes. Front. Immunol..

[330] Al-Haideri M., Tondok S.B., Safa S.H., Maleki A.H., Rostami S., Jalil A.T., Al-Gazally M.E., Alsaikhan F., Rizaev J.A., Mohammad T.A.M. (2022). CAR-T cell combination therapy: The next revolution in cancer treatment. Cancer Cell Int..

[331] Uslu U., Castelli S., June C.H. (2024). CAR T cell combination therapies to treat cancer. Cancer Cell.

[332] Liu Y.E., Wang Y., Yang Y., Weng L., Wu Q., Zhang J., Zhao P., Fang L., Shi Y., Wang P. (2023). Emerging phagocytosis checkpoints in cancer immunotherapy. Signal Transduct. Target. Ther..

[333] Veillette A., Li J., Galindo C.C., Davidson D., Tang Z. (2026). Targeting phagocytosis checkpoints for cancer immunotherapy. Nat. Rev. Cancer.

[334] Chen Y., Zhu X., Chen Y., Yang Z., Shen Z., Chen M., Liu C., Zhou Y., Wang H., Zhu M. (2025). IL-21 promotes the anti-tumor effect of anti-CD47 chimeric antigen receptor macrophages in ovarian cancer. Commun. Biol..

[335] Spolski R., Leonard W.J. (2014). Interleukin-21: A double-edged sword with therapeutic potential. Nat. Rev. Drug Discov..

[336] Wu H., Cheng Y.-P., Liu Y.-L., Zhu R.-H., Ji L.-W., Wang M.-K., Li Q.-X., Shen Z.-L., Ying T.-L., Wu Y.-L. (2026). An engineered IL-21 variant is a potent antitumor candidate for antitumor immunotherapy. Acta Pharmacol. Sin..

[337] Netea M.G., Quintin J., van der Meer J.W. (2011). Trained immunity: A memory for innate host defense. Cell Host Microbe.

[338] Fanucchi S., Domínguez-Andrés J., Joosten L.A.B., Netea M.G., Mhlanga M.M. (2021). The Intersection of Epigenetics and Metabolism in Trained Immunity. Immunity.

[339] Priem B., van Leent M.M.T., Teunissen A.J.P., Sofias A.M., Mourits V.P., Willemsen L., Klein E.D., Oosterwijk R.S., Meerwaldt A.E., Munitz J. (2020). Trained Immunity-Promoting Nanobiologic Therapy Suppresses Tumor Growth and Potentiates Checkpoint Inhibition. Cell.

